# Overexpression of *Bacillus circulans* alkaline protease in *Bacillus subtilis* and its potential application for recovery of protein from soybean dregs

**DOI:** 10.3389/fmicb.2022.968439

**Published:** 2022-08-26

**Authors:** Hao Chen, Jie Wu, Xiaodan Huang, Xuzhong Feng, Hongwu Ji, Liangzhong Zhao, Jianrong Wang

**Affiliations:** ^1^College of Food and Chemical Engineering, Shaoyang University, Shaoyang, China; ^2^Hunan Provincial Key Laboratory of Soybean Products Processing and Safety Control, Shaoyang, China; ^3^College of Food Science and Technology, Guangdong Ocean University, Zhanjiang, China; ^4^Guangdong Provincial Key Laboratory of Aquatic Product Processing and Safety, Zhanjiang, China; ^5^Shenzhen Shanggutang Food Development Co., Ltd.,Shenzhen, China; ^6^Shenzhen Raink Ecology and Environment Co., Ltd.,Shenzhen, China

**Keywords:** alkaline protease, overexpression, *Bacillus circulans*, *Bacillus subtilis*, soybean dregs

## Abstract

Proteases are important for decomposition of proteins to generate peptides or amino acids and have a broad range of applications in different industries. Herein, a gene encoding an alkaline protease (AprBcp) from *Bacillus circulans* R1 was cloned and bioinformatics analyzed. In addition, a series of strategies were applied to achieve high-level expression of AprBcp in *Bacillus subtilis*. The maximum activity of AprBcp reached 165,870 U/ml after 60 h fed-batch cultivation in 50 l bioreactor. The purified recombinant AprBcp exhibited maximum activity at 60°C and pH 10.0, and remained stable in the range from pH 8.0 to 11.0 and 30 to 45°C. Metal ions Ca^2+^, Mn^2+^, and Mg^2+^ could improve the stability of AprBcp. Furthermore, the recombinant AprBcp displayed great potential application on the recovery of protein from soybean dregs. The results of this study will provide an effective method to prepare AprBcp in *B. subtilis* and its potential application on utilization of soybean dregs.

## Introduction

Proteases could decompose proteins to form small peptides or amino acids by cleaving the peptide bonds (Razzaq et al., 2019; Cheng et al., 2021). Proteases are found widely in different sources and mainly from animals, plants, and microorganisms (Ding et al., 2020). Compared with animals and plants, microorganisms are more suitable for production of proteases due to their easy cultivation, extracellular secretion, and excellent properties for industrial application (Contesini et al., 2018). Among different microorganisms, *Bacillus* sp. is the prominent producer for preparation of different proteases, some of which have been commercially applied in food, detergent, feed, and leather industry (Baweja et al., 2016; Contesini et al., 2018). According to the previous studies, proteases from *Bacillus* sp. are mainly composed of alkaline and neutral protease, especially alkaline protease which exhibits maximum relative activity in the range from pH 8.0 to 11.0 (Solanki et al., 2021). Owning to high activity and stability in the range of pH 8.0–11.0, alkaline protease is suitable for application in areas with alkaline conditions such as detergent, leather, and recovery of protein from food-byproduct (Barzkar, 2020; Matkawala et al., 2021). Therefore, many researchers are dedicated to isolation and characterization of alkaline protease from different *Bacillus*. Until now, many alkaline proteases have been isolated and characterized from *Bacillus* (Contesini et al., 2018). However, the expression level of some alkaline protease from wild-type strain is beneath the requirements of industry application. Thus, it is crucial to develop an effective way to obtain high production of alkaline protease.

As a mature expression host, *B. subtilis* is widely used to produce a variety of enzymes and proteins due to its extracellular secretion of target recombinant protein (Song et al., 2015; Cui et al., 2018). Compared with *E. coli* and *Pichia pastoris*, *B. subtilis* exhibits several advantages such as ease of genetic manipulation, being classified as generally recognized as safe (GRAS) and easy cultivation, and short fermentation time (Gu et al., 2018; Wang et al., 2021b). Recently, several proteases are heterologously expressed in *B. subtilis* including alkaline and neutral proteases from *Bacillus clausii* TCCC1004, *Geobacillus stearothermophilus* (*G. stearothermophilus*), *Bacillus licheniformis*, and *Idiomarina* sp. C9-1 (Liu et al., 2013, 2019; Zhou et al., 2018; Chang et al., 2021). However, the extracellular production levels of heterologous proteases in *B. subtilis* vary greatly with respect to proteases from different sources. It is particularly noteworthy that promoter and signal peptides play a pivotal role on the expression levels of heterologous protein in *B. subtilis* (Zhang et al., 2017; Cai et al., 2019; Guo et al., 2021). A series of previous researches demonstrated that the expression level of recombinant protein is improved by 1.1- to 15.2-fold through promoter and signal peptide optimization (Song et al., 2017; Zhang et al., 2017; Fu et al., 2018; Liu et al., 2019; Yao et al., 2019; Kang et al., 2020; Guo et al., 2021). Interestingly, the results of those studies indicated that the strength of promoter and secretion efficiency of signal peptide vary greatly in accordance with the recombinant protein from different microbial sources. For instance, a promoter P_shuttle-09_ from *B. licheniformis* exhibits 8-fold strength than promoter P_43_ for expression of galactosidase in *B. subtilis* (Yang et al., 2013). Nevertheless, the production levels of alkaline proteases from *B. clausii* TCCC1004 in *B. subtilis* are almost same in *B. subtilis* when driving by promoter P_43_ and P_shuttle-09_ (Liu et al., 2019). Similarly, the secretion efficiency of the same signal peptide varies differently according to different recombinant protein in *B. subtilis* (Guan et al., 2016; Zhang et al., 2016b; Liu et al., 2019; Kang et al., 2020). Based on the above-mentioned results, it is concluded that it is necessary to optimize promoter or signal peptide to achieve high-level expression of each target protein. Analysis of previous studies we found that plasmid-based expression is the most favorite choice for heterologous expression of recombinant protein in *B. subtilis*. However, plasmid-based expression has some disadvantages such as unstable in medium without antibiotic, and vectors with antibiotic resistances, which limit its further industrial application (Yang et al., 2016; Chen et al., 2017). Compared with plasmid-based expression, integrative approach expression of recombinant protein seems more suitable for industrial application due to its stable and no antibiotic resistance-encoding gene (Zhou et al., 2021). Additionally, integrative approach expression is convenient for further genetic manipulation of recombinant strain (Zhou et al., 2020, 2021).

Soybean dregs, also known as okara, are the by-product of soybean products, with about 2.8 million tones annually produced in China (Li et al., 2012). Although the soybean products mainly make use of the protein in soybean, there is still a lot left in soybean dregs. Many studies have shown that protein accounts for 15.2–37.5% of dry weight of soybean dregs and contains all essential amino acids necessary for good health (Li et al., 2012; Fayaz et al., 2019). So far, soybean dregs mainly are discarded as waste, with a small amount used as animal feed or fertilizers, resulting in environmental pollution and waste resources. Therefore, soybean dregs can be consumed as an underutilized and cheap plant protein resource for human consumption. To date, several methods have been used to recover protein from soybean dregs such as fermentation, chemical, and enzymatic methods (Cao et al., 2019; Wang et al., 2021a; Heng et al., 2022). Among the above-mentioned methods, the enzymatic method has attracted more attention due to its mild reaction conditions and environmental friendliness. As the key factor of enzymatic method, protease could hydrolyze peptide bonds to produce small peptides, and then, recover most of the protein from soybean dregs.

In this study, the gene encodes an alkaline protease from *B. circulans* R1 (named AprBcp) was cloned and bioinformatics analyzed. Meanwhile, the promoters and signal peptides were optimized to drive the heterologous expression of AprBcp in *B. subtilis*. Moreover, integrative approach and large-scale cultivation were carried out to achieve high-level production of AprBcp in *B. subtilis*. Additionally, the recombinant AprBcp was purified and characterized. Finally, the application of recombinant AprBcp for recovery of protein from soybean dregs was investigated. The results of this study will provide an effective method to produce AprBcp in *B. subtilis* and its potential application on utilization of soybean dregs.

## Materials and methods

### Strains, reagents, and medium

The *Escherichia coli* strain top 10 was used to propagate plasmids. *B. circulans* strain R1 was isolated from Okara and conserved in our laboratory. The *B. subtilis* strains RIK1285, pMD20T, and in-fusion cloning kit were purchased from Takara Biotechnology (Dalian, China). Q5® high-fidelity DNA polymerase and protein marker were purchased from New England Biolabs (NEB, Ipswich, MA, United States). Different inhibitors, such as pepstatin A, iodoacetamide, ethylenedia minetetraacetic acid (EDTA), and phenylmethanesulfonyl fluoride (PMSF), and substrates, such as casein, bovine serum albumin (BSA), keratin, soybean protein isolate, and chicken egg albumin, were purchased from Yuanye Biotechnology (Shanghai, China). Soybean dregs were by-products of manufacturing fermented soybean whey-based tofu, dried by microwave drying, ground, and sieved with 60 mesh. The soybean dregs comprised 22.03% protein and 10.23% water.

LBK (LB with 30 μg/ml kanamycin) was used to screen *E. coli* top 10 and *B. subtilis* RIK1285 contained plasmids with kanamycin-resistant gene (Kan^R^). Fermentation medium 1 was used for the production of AprBcp in 24-well plates and shake flask, which was composed of 1.5% peptone, 2.5% yeast extract, 1% citrate sodium, 0.3% K_2_HPO_4_, and 4% maltose. Fermentation medium 2 was used for large-scale production of AprBcp in 7 and 50 l bioreactor, which contained 7% maltose, 2.5% soybean meal, 2% corn steep liquor, 0.3% citrate sodium, 1% K_2_HPO_4_, 2% wheat bran, and 0.3% CaCl_2_.

### Cultivation conditions

The 24-well plate cultivation was used to screen the recombinant strains with different promoters and signal peptides. The shake flask cultivation was used to isolate the recombinant strains with higher alkaline protease activity. Large-scale production of AprBcp was carried out in 7- and 50-L bioreactor. The detailed protocol is provided in Supplementary Methods.

### Gene cloning and bioinformatics analysis

Based on the sequence of alkaline protease gene from *B. circulans* strain NCTC2610 (GenBank: UAPZ01000012.1: 120935-122077), two primers, *bcp*-fw and *bcp*-rev, were designed for PCR amplification. The gene encoding AprBcp was cloned by PCR amplification using genomic DNA of *B. circulans* R1 as template. The PCR product was ligated to pMD20T and sequenced. The primers used in this study are shown in Supplementary Table 1.

Sequence alignment was performed by blastn or blastx provided by the National Center for Biotechnology Information (NCBI). Amino acid sequences and signal peptide prediction of AprBcp were analyzed by Expasy.^1^ The sequences were analyzed for identity using ClustalW^2^ and ENDscript/ESPript.^3^ Homologous models were generated on the Swiss-model online server. PyMOL was used to analyze the obtained model. The alkaline serine protease from *B. lentus* was chosen as a template (Protein Data Bank code: 1st3.1.A).

### Promoter optimization

**Figure 1:**
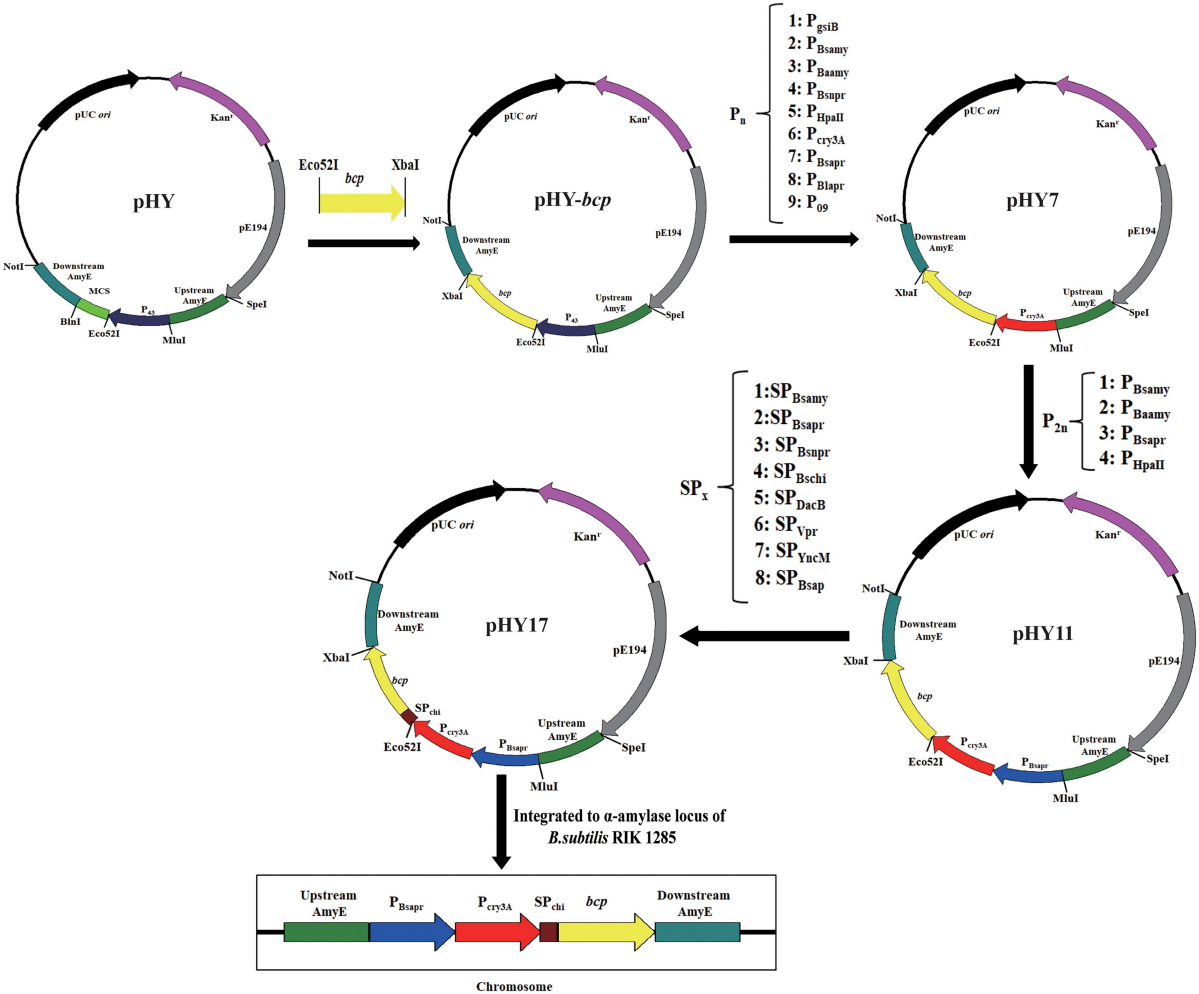
Sketch of the principal procedures for plasmids and strain engineering.

The vector pHY was constructed as template to construct different plasmids (Figure 1). The vector pHY was composed of temperature-sensitive replicon PE194 and a kanamycin-resistant gene (Kan^R^) which functions in *B. subtilis* and *E. coli*, as well as the pUC origin of replication from vector pBluescript IISK(+). Additionally, it included *B. subtilis*-derived promoter P_43_, which located upstream from the multi-cloning site (MCS), the 5′-upstream repair, and 3′-downstream repair template of α-amylase from *B. subtilis* 168.

The gene *bcp* was ligated to pHY to form expression vector pHY-*bcp*. For promoter optimization, nine highly active promoters reported by previous studies were selected and synthesized for heterologous expression of AprBcp. The fragment pHY without promoter P_43_ was obtained from pHY-*bcp* digested by *Eco52*I and *Mlu*I. And then, each different promoter flanked by *Eco52*I-MluI was separately ligated to fragment pHY to form nine expression plasmids. For dual promoter plasmids, the second promoter was inserted into the upstream of the single promoter expression plasmid (Figure 1). The sequences of nine highly active promoters are shown in Supplementary Table 1 and the primers used in this part are shown in Supplementary Table 1.

### Signal peptide optimization

For signal peptide optimization, the expression vector with the highly active dual promoters was used to screen the efficient signal peptide. Nine signal peptides were selected and synthesized by over-lapping PCR, and ligated to dual promoter expression plasmid by in-fusion cloning. The process for construction of plasmids with signal peptides is shown in Figure 1. Plasmids with different promoters and signal peptides used in this study are shown in Table 1. The sequences of nine signal peptides are shown in Supplementary Table 3 and the primers used in this part are also shown in Supplementary Table 1.

**Table 1:** Bacterial strains and plasmids used in this study.

Strains	Plasmids	Promoter	Signal peptide	Host
Ec1	pMD20T	None	None	*E. coli* top 10
Bs1	pHY1	P_43_	SP_bcp_	*B. subtilis* 1285
Bs2	pHY2	P_gsiB_	SP_bcp_	*B. subtilis* 1285
Bs3	pHY3	P_Bsamy_	SP_bcp_	*B. subtilis* 1285
Bs4	pHY4	P_Baamy_	SP_bcp_	*B. subtilis* 1285
Bs5	pHY5	P_Bsnpr_	SP_bcp_	*B. subtilis* 1285
Bs6	pHY6	P_HpaII_	SP_bcp_	*B. subtilis* 1285
Bs7	pHY7	P_cry3A_	SP_bcp_	*B. subtilis* 1285
Bs8	pHY8	P_Bsapr_	SP_bcp_	*B. subtilis* 1285
Bs9	pHY9	P_Blapr_	SP_bcp_	*B. subtilis* 1285
Bs10	pHY10	P_09_	SP_bcp_	*B. subtilis* 1285
Bs11	pHY11	P_Bsapr-cry3A_	SP_bcp_	*B. subtilis* 1285
Bs12	pHY12	P_Baamy-cry3A_	SP_bcp_	*B. subtilis* 1285
Bs13	pHY13	P_Bsamy-cry3A_	SP_bcp_	*B. subtilis* 1285
Bs14	pHY14	P_HpaII-cry3A_	SP_bcp_	*B. subtilis* 1285
Bs15	pHY15	P_Bsapr-cry3A_	SP_Bsamy_	*B. subtilis* 1285
Bs16	pHY16	P_Bsapr-cry3A_	SP_Bsapr_	*B. subtilis* 1285
Bs17	pHY17	P_Bsapr-cry3A_	SP_Bschi_	*B. subtilis* 1285
Bs18	pHY18	P_Bsapr-cry3A_	SP_Bsnpr_	*B. subtilis* 1285
Bs19	pHY19	P_Bsapr-cry3A_	SP_DacB_	*B. subtilis* 1285
Bs20	pHY20	P_Bsapr-cry3A_	SP_Vpr_	*B. subtilis* 1285
Bs21	pHY21	P_Bsapr-cry3A_	SP_YncM_	*B. subtilis* 1285
Bs22	pHY22	P_Bsapr-cry3A_	SP_Bsap_	*B. subtilis* 1285
Bs23 to Bs32	None	P_Bsapr-cry3A_	SP_Bschi_	*B. subtilis* 1285

### Mark-free and large-scale production of AprBcp

**Figure 2:**
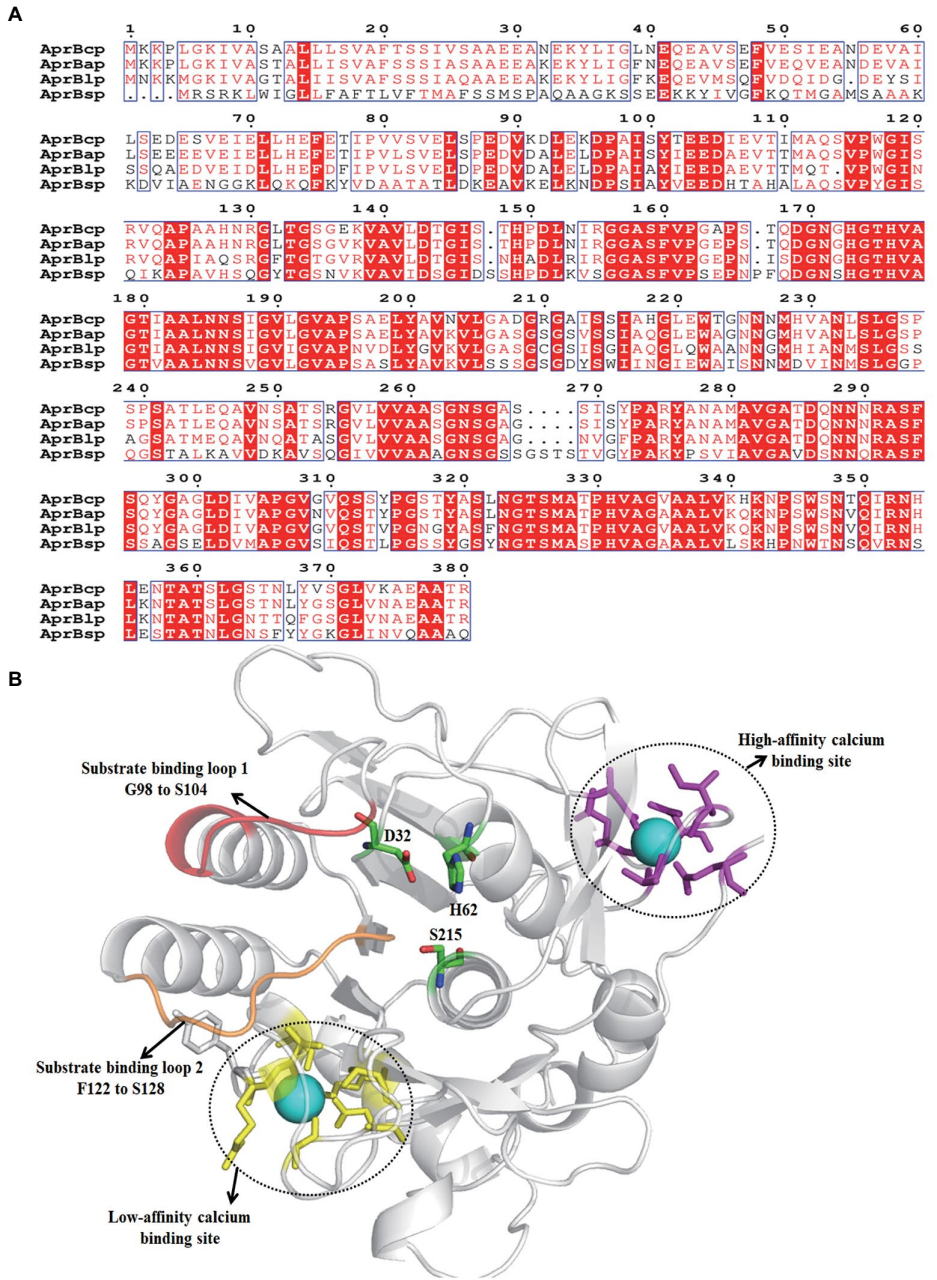
Sequence analysis of AprBcp. **(A)** Sequence alignment of AprBcp with other alkaline proteases. The listed sequences included the alkaline protease AprBlp from *Bacillus lehensis* (AFK0897 0.1), AprBap from *Bacillus alcalophilus* (FJ940727.1) and AprBsp from *B. subtilis* (WP_003327717.1). **(B)** Homology modeling structure of AprBcp.

The *bcp* expression cassette, with highly active dual promoter, efficient signal peptide and *bcp*, was integrated to α-amylase locus of *B. subtilis* 1285. The method for chromosomal integration of *bcp* expression cassette was similar to previous studies and the detailed process is provided in Supplementary Methods and Figure 2 (Zakataeva et al., 2010; Zhang et al., 2016a; Zhou et al., 2021). The recombinant strains with *bcp* expression cassette integrated to α-amylase locus were cultivated in a 500 ml flask for 48 h. The recombinant strain with the highest activity at 500 ml shake flask was further cultivated in 7 and 50 l bioreactor, respectively.

### Purification and enzyme assay

The fermentation supernatant after centrifugation was filtered through with a 0.22 μm membrane, then concentrated and buffer-exchanged to Tris–HCl buffer (20 mM, pH 7.5) by ultrafiltration with a membrane of 10 kDa cutoff. The AprBcp was purified by HiTrap SP Fast Flow chromatography. The purified AprBcp was collected and analyzed by 15% sodium dodecyl sulfate-polyacrylamide gel electrophoresis (SDS-PAGE).

The protease activity was detected according to the Folin’s phenol method (Wang et al., 2019). A quantity of 1 g casein was dissolved in 100 ml boric acid buffer (pH 10.0, 50 mM) and used as substrate. Firstly, 200 μl of diluted enzyme was added to 200 μl of 1% (w/v) casein solution and incubated at 60°C for 10 min. Secondly, 400 μl of 400 mM trichloroacetic acid (TCA) was added to end the reaction, and then centrifuged at 11,000 × *g* for 5 min. Finally, 200 μl of the supernatant was added into 1 ml of 400 mM Na_2_CO_3_ and 200 μl of Folin’s phenol reagent and incubated at 40°C for 20 min. The reactions with enzymes added after TCA were used as controls. One unit of alkaline protease activity was defined as the amount of enzyme that releases 1 μg of tyrosine from casein at 60°C and pH 10.0.

### Characterization of AprBcp

The optimal pH of AprBcp was assayed in different 50 mM buffer from pH 6.0 to 11.0 (Na_2_HPO_4_–NaH_2_PO_4_ for pH 6.0–7.0, Tris–HCl for pH 8.0–9.0, and boric acid-NaOH for pH 10.0–11.0). The relative activity at different pH was calculated by setting pH 10.0 as 100%. For the pH stability, AprBcp was incubated at 30°C in 50 mM buffer with different pH from 6.0 to 11.0 for 6 h, and then the residual activity was determined. The highest enzyme activity was considered as 100%, and other values were recorded as a percentage of the highest value.

The optimal temperature of AprBcp was measured at different temperatures from 40°C to 75°C. The relative activity at different temperatures was calculated by setting 60°C as 100%. The thermal stability of AprBcp was investigated by incubating at temperatures from 40°C to 70°C for 10 min, and the residual activity was determined at pH 10.0 and 60°C. Furthermore, thermal stabilities of AprBcp at 60°C in presence of 2, 5, and 10 mM Ca^2+^ were determined by incubation at different time intervals. The residual activity was calculated by taking the non-heated AprBcp activity as 100%.

### Effects of inhibitors on the activity of AprBcp

Several inhibitors, such as pepstatin A, PMSF, EDTA, and iodoacetamide, were used to investigate the inhibition effects on the activity of AprBcp. The residual activity of AprBcp was detected in presence of these inhibitors and calculated by taking the non-treated AprBcp activity as 100%.

### Effects of metal ions on the stability of AprBcp

The effect of different metal ions on the activity of AprBcp was analyzed by incubating enzyme samples for 6 h at room temperature in 50 mM Tris–HCl buffer (pH 8.0), containing 1 and 5 mM of Ca^2+^, Mg^2+^, Na^+^, K^+^, Li^+^, Zn^2+^, Mn^2+^, Co^2+^, Hg^2+^, Ag^+^, and Cu^2+^. The residual activity was determined as described previously.

### Substrate specificity profile of AprBcp

For substrate specificity, casein, BSA, keratin, soy protein isolate, and chicken egg albumin were used as substrate, respectively. The relative activity on each substrate was expressed as the percentage of that on casein.

### Recovery of protein from soybean dregs by AprBcp

The soybean dregs powders were mixed with deionized water at a ratio of 3% (w/w), and homogenized using a homogenizer at a speed of 11,000 × *g* for 3 min before it was adjusted pH to 9.0 with 1 M NaOH. The mixtures were gradually raised to 50°C prior to the addition of proteinase AprBcp, and then continuously shook at that temperature for 3 h. The resulting suspensions were incubated in boiling water for 10 min to inactivate the proteinase, and subsequently centrifuged at 11,000 × *g* for 30 min to remove the insoluble residue. The collected supernatant was acidified with 3 M HCl to pH 7.0.

Different ratios of enzyme to substrate (500, 1,000, 1,500, 2,000, and 2,500 U/g), ratio of substrate to water (1, 2, 3, 4, and 5%, w/w), hydrolysis time (2, 3, 4, 5, and 6 h), hydrolysis temperature (40, 45, 50, 55, and 60°C), and pH (7, 8, 9, 10, and 11) were selected successively for optimization.

The protein recovery (RP, %) was used as a measurement of the soybean dregs’ protein extraction efficiency and defined as the ratio of the protein mass in the supernatant to that in the soybean dregs. The protein content was determined using the GB 5009.5-2016 of the Chinese standard.

### Statistical analysis

All measurements were done in triplicate. The data were analyzed by the Statistical Package for the Social Sciences (SPSS) software (version 18.0, SPSS Inc., United States) and were expressed as mean ± standard deviation (SD).

## Results and discussion

### Bioinformatics analysis

The results of NCBI-blastn and open reading frame finder exhibited that the open reading frame of *bcp* was 1,143 bp, which encoded a polypeptide with 381 amino acid residues. The sequence of *bcp* was deposited in the GenBank of NCBI (accession no. ON733249). Based on the results of NCBI-blastp, we deduced that AprBcp is an alkaline serine protease, which belonged to S8 family peptidase. In addition, AprBcp displayed 92.3, 89.4, and 78.7% identity against alkaline serine protease from *B. circulans* NCTC2610 (accession no. SPU21234.1), *Alkalihalobacillus clausii* (accession no. WP_094423791), and *Alkalihalobacillus patagoniensis* (accession no. WP_09419032 9.1), respectively. Multiple alignment analyses of different alkaline serine protease with AprBcp displayed that alkaline proteases from *bacillus* are composed of three different domains, which include signal peptide, pro-peptide, and mature peptide domain (Figure 2A). For AprBcp, the first 27 amino acid residues, the sequence from Ala28 to Met111, and the sequence from Ala112 to Arg380 were signal peptide, pro-peptide, and mature peptide domain, respectively.

The tertiary structure of AprBcp with mature peptide domain was obtained by homology modeling using the alkaline proteases from *Bacillus lentus* as a template (PDB deposition: 1st3.1.A). As shown in Figure 2B, the catalytic triad consisted of D32, H62, and S215. In addition, two surface loops, located from position G98 to S104 and F122 to S128, were responsible for substrate binding. Similar to already reported alkaline proteases (Zhang et al., 2019), AprBcp also has two calcium-binding sites. The first calcium-binding site with high affinity is involved of a loop from position A72 to L80 and side chains of Gln2 and Asp40. The second calcium-binding site, a low-affinity binding site, was formed by two bound water and residues Tyr165, Ala168 Asp191, and Arg241.

### Promoter optimization

**Figure 3:**
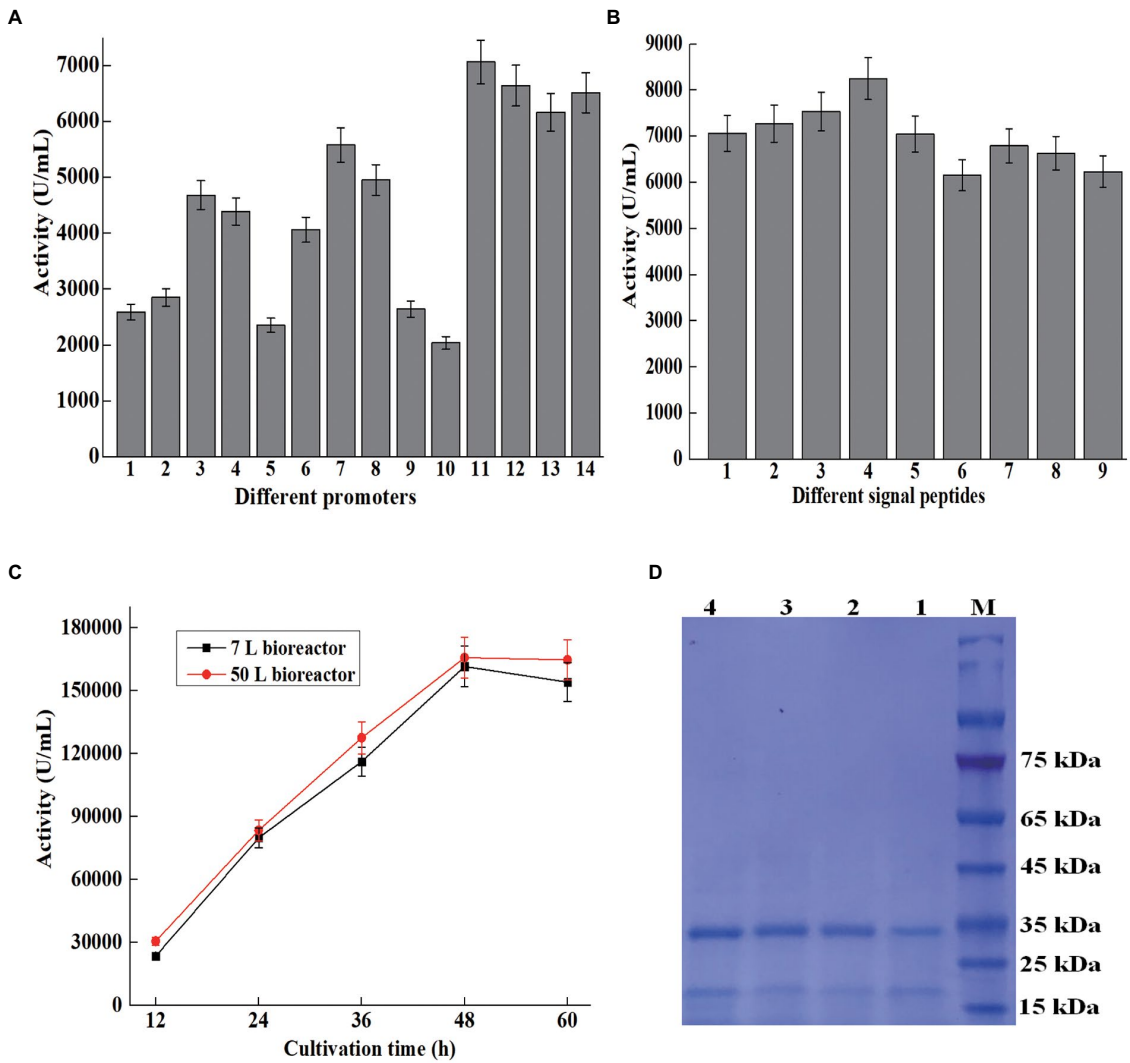
Overexpression of AprBcp in *Bacillus subtilis*. **(A)** Promoter optimization. Numbers 1–14 represent promoter P_43_, P_gsiB_, P_Bsamy_, P_Baamy_, P_Bsnpr_, P_HpaII_, P_cry3A_, P_Bsapr_, P_Blapr_, P_09_, P_Bsapr-cry3A_, P_Baamy-cry3A_, P_Bsamy-cry3A_, and P_HpaII-cry3A_. **(B)** Signal peptide optimization. Numbers 1–9 represent signal peptide SP_bcp_, SP_Bsamy_, SP_Bsapr_, SP_Bschi_, SP_Bsnpr_, SP_DacB_, SP_Vpr_, SP_YncM_, and SP_Bsap_. **(C)** Large-scale production of AprBcp in 7 and 50 l bioreactor. **(D)** SDS-PAGE analysis of supernatant from different cultivation time in 50 l bioreactor. Numbers 1–4 represent supernatant from 12 to 60 h.

In this study, 10 promoters were chosen to drive the expression of AprBcp in *B. subtilis* 1285 (Table 1). The process for construction of expression vectors with different promoters was depicted in Figure 1. The obtained 10 expression vectors were transformed to *B. subtilis* 1285 and the strengths of those 10 promoters were evaluated by extracellular activity of AprBcp. As exhibited in Supplementary Figure 2 and Figure 3A, the recombinant strain Bs7 containing plasmid pHY7 with promoter P_cry3A_ exhibited the highest activity at both 24-well plate and shake flask cultivation, followed by Bs8 (P_Bsapr_), Bs3 (P_Bsamy_), Bs4 (P_Baamy_), and Bs6 (P_HpaII_). For cultivated 500 ml shake flask, the highest activity of Bs7 was 5,580 U/ml, which is 1.13- to 2.73-fold higher than that of another recombinant strain containing different promoters (Figure 3A). Until now, there are few reports showed that promoter P_cry3A_ from *Bacillus thuringiensis* could drive high-level expression of alkaline protease in *B. subtilis* (Widner et al., 2000). The results of this study demonstrated that promoter P_cry3A_ was more suitable for heterologous expression of recombinant AprBcp in *B. subtilis* 1285 than another nine promoters. Our results were similar to previous studies, which demonstrated that optimization of promoter could effectively improve the production of recombinant protein in *B. subtilis* (Song et al., 2015; Song et al., 2017; Zhang et al., 2017; Liu et al., 2019; Kang et al., 2020).

As a key regulatory element, promoter plays an important role on the expression level of heterologous gene in *B. subtilis*. Many studies demonstrated that the strength of dual promoter was normally higher than the single in *B. subtilis* (Guan et al., 2016; Zhang et al., 2017; Liu et al., 2019; Kang et al., 2020). For further improvement of the production of AprBcp, expression vectors with dual promoter were constructed (Table 1; Figure 1). According to previous work, the transcriptional strength of a dual promoter depends on the promoter adjacent to the gene, so promoter P_cry3A_ was located adjacent to *bcp*. Promoter P_Bsapr_, P_Bsamy_, P_Baamy_, and P_HpaII_ were inserted to pHY7 upstream P_cry3A_, generating plasmids with dual promoter such as P_Bsapr-cry3A_, P_Baamy-cry3A_, P_Bsamy-cry3A_, and P_HpaII-cry3A_, which were in accordance with plasmids pHY11, pHY12, pHY13, and pHY14, respectively. As exhibited in Supplementary Figure2 and Figure 3A, the extracellular activities of recombinant strains with dual promoter were higher than Bs7 (P_cry3A_), which demonstrated that dual promoter could further improve the production of recombinant AprBcp. Among those recombinant strains, Bs11 (P_Bsapr-cry3A_) with highest extracellular activity (7,068 U/ml), which was 1.26-fold higher than that of Bs7 (P_cry3A_). According to the previous literatures, two tandem promoters can improve the production of recombinant protein in *B. subtilis*. As reported by Kang et al. (2020), the production of recombinant amidase drove by dual promoter P_amyE-cdd_ was 1.37-fold higher than that of the control. Similarly, the activity of extracellular BcaPRO from recombinant strain containing dual promoter P_Bsamy-Baamy_ was 1.5-fold of the activity of strain containing single promoter P_Baamy_ (Liu et al., 2019). In recent years, different dual tandem promoters have been reported to drive expression of recombinant protein in *B. subtilis* such as P_Bsamy-Baamy_, P_amyE-cdd_, P_spoVG-spoVG142_, P_HpaII-amyQ_, and P_gsiB-HpaII_ (Guan et al., 2016; Zhang et al., 2017; Liu et al., 2019; Kang et al., 2020). To our best knowledge, there are no reports about dual promoter P_Bsapr-cry3A_. The results of this study revealed that dual tandem promoter P_Bsapr-cry3A_ with great potential for heterologous expression of recombinant protein in *B. subtilis.*

### Signal peptide optimization

As a key regulatory element, signal peptide plays an important role on efficient extracellular expression of target protein in *B. subtilis* (Song et al., 2017; Fu et al., 2018). To further improve the production of AprBcp in *B. subtilis*, eight efficient sec-type signal peptides from *B. subtilis* were selected to replace the native signal peptide of AprBcp (Table 1; Figure 1). As shown in Supplementary Figure 3 and Figure 3B, the recombinant strain BS17 (containing signal peptide SP_Bschi_) exhibited the highest activity among those eight recombinant strains containing different signal peptides. The maximum extracellular activity of recombinant strain BS17 was 8,253 U/ml, which was almost about 1.09- to 1.33-fold higher than that of the other strain with different signal peptides. Until now, there are few studies reported the signal peptide SP_Bschi_ of chitinase from *B. subtilis*. Generally, signal peptides of amylase and protease from *B. subtilis*, *B. licheniformis,* or *Bacillus amyloliquefaciens* are usually used to the secretion of recombinant protein in *B. subtilis.* The result of this study revealed that signal peptide SP_Bschi_ of chitinase is more efficient than that from α-amylase, alkaline, and neutral protease.

Generally, the signal peptide is composed of positively charged N-region, hydrophobic H-region, and C-region. The positively charged N-region is responsible for the interaction with the translocation machinery. Additionally, the hydrophobic H-region, following the charged N-region, functions on the process of insertion of signal peptide into the membrane. Previous studies indicated that the efficiency of signal peptide is related to its positively charged N-region and hydrophobic H-region (Guo et al., 2021). In this study, the N-domain charge and the ratio of hydrophobic amino acids of eight efficient Sec-type signal peptides used in this study were from 2 to 4, and 54.5 to 72.7%, respectively (Supplementary Table 4). The most efficient signal peptide SP_Bschi_ with the highest N-domain charge, but lowest ratio of hydrophobic amino acids, which indicated that the efficiency of secretion may not direct relative to the properties described above. Based on the results of previous (Liu et al., 2019; Kang et al., 2020) and this study, we concluded that each target protein has its preference for signal peptide and it is necessary to screen a suitable signal peptide for efficient extracellular production of target protein.

### Mark-free expression and large-scale production of AprBcp

During the processes of promoter and signal peptide optimization, the expression of AprBcp was mediated by free plasmids, and the kanamycin was required to maintain expression vectors. However, cultivation added with kanamycin and the plasmid with kanamycin resistance gene marker are both prohibited in many application industries (Yang et al., 2016). Therefore, it is necessary to develop a mark-free expression way to produce AprBcp. The expression vector pHY17 contains temperature-sensitive replicon PE194, which will be inactivated when cultivation temperature is up to 45°C. Then, the fragment with dual promoter P_Bsapr-cry3A_, signal peptide SP_Bschi_, *bcp*, 5′-upstream and 3′-downstream homologous template of α-amylase will be integrated to α-amylase locus of *B. subtilis*1285. A total of 10 mark-free strains named Bs23 to Bs32 were selected and cultivated in shake flask. The extracellular activities of these 10 mark-free strains were from 8,123 to 8,365 U/ml and the strain Bs27 with highest activity (8,365 U/ml) was chosen for large-scale fermentation.

The large-scale fermentation of Bs27 was carried in 7- and 50-L bioreactor. The maximum activity produced by recombinant strain Bs27 in 7- and 50-L bioreactor were 161,661 and 165,870 U/ml, respectively (Figure 3C). In recent years, several engineered *Bacillus* strains, such as *B. subtilis*, *B. licheniformis*, and *B. amyloliquefaciens*, were cultivated in a bioreactor to prepare alkaline protease (Liu et al., 2019; Wang et al., 2019; Zhou et al., 2020, 2021). Previous studies reported that the extracellular alkaline protease activity of engineered *B. licheniformis* 2709, *B. amyloliquefaciens* K11, and *B. subtilis* WB600 reached 57,763, 30,200, and 27,860 U/ml, respectively, in 5 or 15 l fermenter (Wang et al., 2016, 2019; Liu et al., 2019; Zhou et al., 2021). However, for most of the studies, the method for alkaline protease activity assay was based on the method of the People’s Republic of China GB/T 23527-2009, in which one unit of alkaline protease activity was defined as the amount of enzyme that releases 1 μg of tyrosine from casein at 40°C and pH 10.5. Thus, our results were not fully comparable with these values since activity assays with different conditions have been used. SDS-PAGE analysis of fermented supernatant from different cultivation times in 50-L bioreactor revealed that AprBcp was approximately free from contaminating proteins, which facilitates downstream processing (Figure 3D).

### Purification and kinetic parameters

The process for purification of AprBcp is shown in Supplementary Table 5. After ultrafiltration and affinity chromatography, AprBcp was purified 2.09-fold to homogeneity and specific activity reached 58,621 U/mg, which was higher than many previous reported alkaline proteases but lower than those from *Idiomarina* sp. C9-1 (99,511.9 U/mg), *Termitomyces albuminosus* (180,000 U/mg), *Trametes cingulata* CTM10101 (94,000 U/mg), and *Vibrio metschnikovii* J1 (120,000 U/mg; Jellouli et al., 2009; Zheng et al., 2011; Omrane Benmrad et al., 2016; Zhou et al., 2018). SDS-PAGE analysis showed that the purified AprBcp was about 30 kDa corresponding to molecular mass of mature peptide of AprBcp, which is similar to other alkaline proteases from *Bacillus* (Contesini et al., 2018). It has been reported that alkaline proteases from *Bacillus* species almost consist of a signal peptide, pro-peptide, and subtilisin domain. Theoretically, alkaline proteases from *Bacillus* are activated by three steps: (1) folding of alkaline protease containing different domains, (2) auto-processing between the pro-peptide and subtilisin domain, and (3) degradation of the pro-peptide by activated subtilisin (Uehara et al., 2013).

The values of *K*_m_ and *V*_max_ of purified AprBcp were 2.35 mg/ml and 63,038 μM min^−1^ ml^−1^, respectively. Kinetic parameters of purified AprBcp indicated that it has high substrate affinity and catalytic efficiency toward casein. Based on previous studies, we found that *K*_m_ values of *Bacillus* alkaline proteases vary differently. The *K*_m_ values of alkaline proteases from *B. cereus*-S6-3, *Bacillus stearothermophilus*, *Bacillus safensis* RH12, and *B. circulans* M34 are 3.07, 0.62, 2.2, and 0.96 mg casein/mL, respectively (Sari et al., 2015; Abdel-Naby et al., 2017, 2020; Rekik et al., 2019).

### Characterization of AprBcp

**Figure 4:**
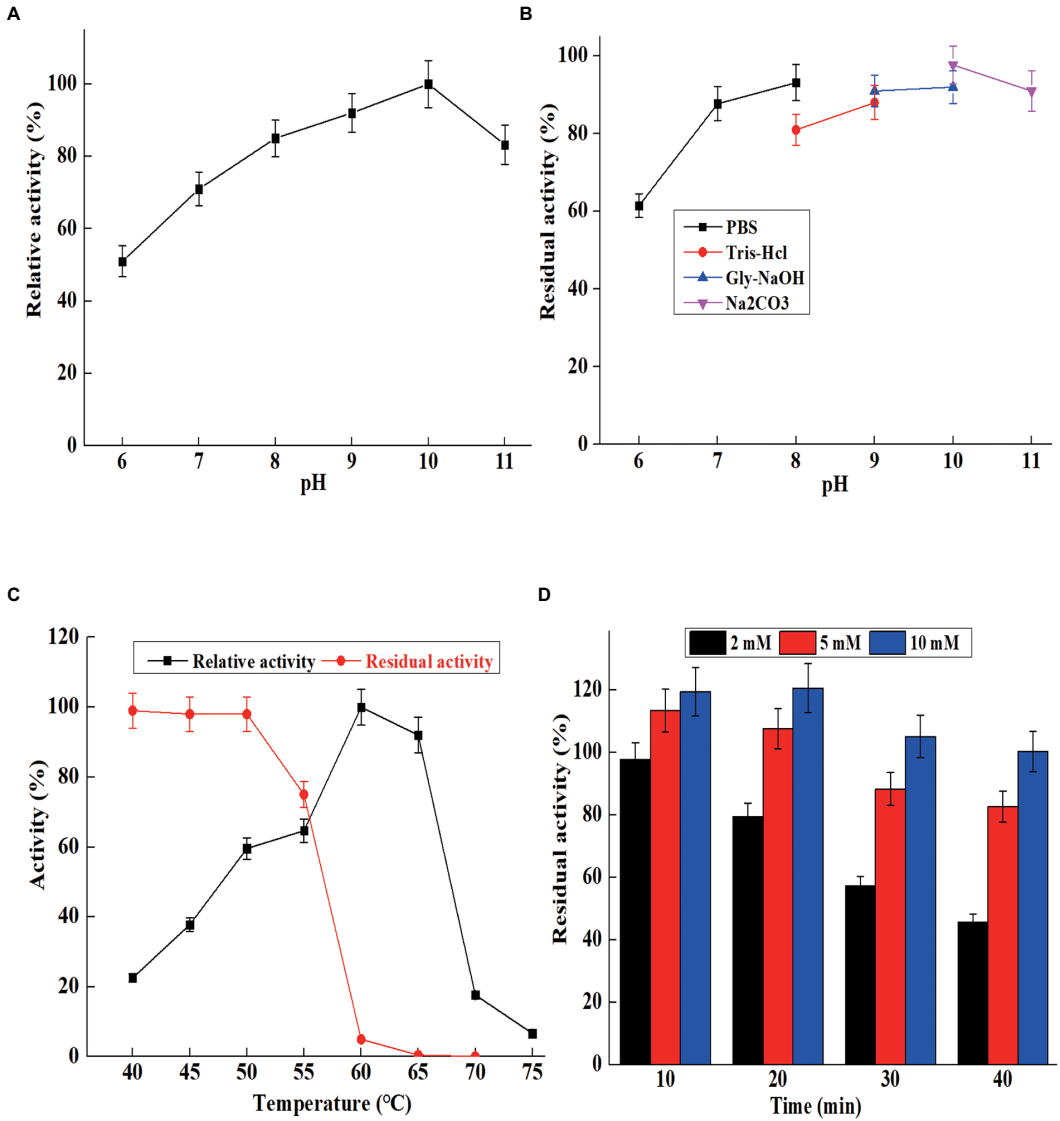
The characterization of purified AprBcp. **(A)** Optimum pH of purified AprBcp. **(B)** pH stability of purified AprBcp. **(C)** Optimum temperature and thermal stability of purified AprBcp. **(D)** The effects of different concentration of Ca^2+^ on the stability of AprBcp incubating at 60°C.

The optimum pH for maximum activity of AprBcp was 10.0, and the relative activities were above 65% in the range from pH 7.0 to 11.0 (Figure 4A). Generally, the most reported alkaline proteases exhibit higher activity in the range of 8.0–11.0. The maximum activities of alkaline proteases from *B. pumilus* AR57 and *Bacillus megaterium* RRM2 are detected at 9.0 and 10.0, respectively (Rajkumar et al., 2011; Jagadeesan et al., 2020). While, the optimal pH of an alkaline protease from *Bacillus halodurans* C-125 is 12.0 (Tekin et al., 2021). For pH stability, AprBcp exhibited high residual activity (above 80%) in the range from pH 8.0 to 11.0 after 5 h of incubating at 25°C (Figure 4B), which is similar to previous reported alkaline protease (Rajkumar et al., 2011; Jagadeesan et al., 2020; Tekin et al., 2021).

For optimum temperature, AprBcp exhibited maximum relative activity at 60°C and remained highly active from 50 to 65°C (Figure 4C). The optimum temperature of already studied alkaline proteases verifies differently according to their origin. Normally, alkaline proteases from *Bacillus* showed high activity in the range from 50 to 80°C (Contesini et al., 2018). As far as thermal stability analysis is concerned, the purified AprBcp was incubated in buffers with and without Ca^2+^. The residual activities of AprBcp in the absence of Ca^2+^ at 50, 55, 60, and 65°C were 98.2, 75.3, 5.2, and 0.5%, respectively, after heat treatment for 10 min (Figure 4C). As shown in Figure 4D, it was obvious that addition with Ca^2+^ could effectively improve the thermal stability of AprBcp. The residual activities of AprBcp in the presence of 2, 5, and 10 mM Ca^2+^ were 45.8, 82.3, and 100.3%, respectively, after heat treatment at 60°C for 40 min. It has been reported that Ca^2+^ plays an important role on the stability of alkaline protease. For instance, the stability of alkaline protease from *Trametes cingulata* CTM10101, *Nocardiopsis alba* OM-5, and *B. safensis* S406 was enhanced by 1.24-to 4.52-fold, respectively (Omrane Benmrad et al., 2016; Mhamdi et al., 2017; Chauhan et al., 2021). According to the results from Zhang et al. (2019), Ca^2+^ could as an activator to improve the stability and activity of alkaline protease from *Bacillus* species is attributed to its electrostatic interaction with alkaline protease. In this study, there were two calcium-binding sites in AprBcp to interact with Ca^2+^, which improved the stability of AprBcp in extreme conditions.

### Effects of inhibitors on the activity of AprBcp

It has been reported that proteases from different sources are sensitive to various inhibitors (Barzkar, 2020). Usually, serine proteases are inhibited by PMSF, diisopropyl fluorophosphate, and 3,4-dichloroisocoumarin. The relative activities of AprBcp treated with different inhibitors are shown in Table 2. AprBcp was strongly inhibited by PMSF and the relative activity was only 27.2 and 0.5%, respectively, in the presence of 1 and 5 mM PMSF, suggesting that serine is relative to the catalytic activity. Based on previous studies, we found that the activities of most serine proteases were completely inhibited by PMSF at a concentration of 5 mM (Barzkar, 2020; Jagadeesan et al., 2020; Chang et al., 2021). However, for an alkaline serine protease from *Pseudoalteromonas* sp. 129–1, the maximum activity inhibition is detected in the presence of 15 mM PMSF (Wu et al., 2015). In addition, the relative activities of AprBcp were 86.3 and 73.2%, respectively, in presence of 1 and 5 mM EDTA, revealing that some metallic ions are important for catalytic activity of AprBcp. The inhibitory effect of EDTA on AprBcp is similar to previous studies (Omrane Benmrad et al., 2018; Sharma et al., 2020; Chang et al., 2021). The alkaline proteases from *Nocardiopsis dassonvillei* OK-18, *B. stearothermophilus* CAU209, and *Penicillium chrysogenum* X5 are inhibited by 30.8, 40.5, and 22.2%, respectively, in presence of 1 to 10 mM EDTA (Omrane Benmrad et al., 2018; Sharma et al., 2020; Chang et al., 2021). Compared with PMSF and EDTA, pepstatin A and iodoacetamide exhibited little inhibitory effects on the activity of AprBcp, which indicated that-SH group and aspartate were not involved in the catalytic activity of AprBcp.

**Table 2:** Effects of inhibitors on the activity of AprBcp.

Inhibitors	Residual activity (%)	
	1 mM	5 mM
PMSF	27.2	0.2
EDTA	86.3	73.2
pepstatin A	98.3	97.6
iodoacetamide	97.3	96.5

### Effects of metal ions on the stability of AprBcp

Several metal ions were assessed for their effects on the stability of AprBcp (Table 3). As activator, metal ions Ca^2+^, Mn^2+^, and Mg^2+^ could improve the residual activity of AprBcp by 115.2, 112.3, and 108.5%, respectively, at a concentration of 5 mM. Besides, AprBcp was inhibited by Cu^2+^ and the residual activities were 40.7 and 25.9%, respectively, in the presence of 1 and 5 mM Cu^2+^. Other metal ions, such as K^+^, Na^+^, Co^2+^, Zn^2+^, and Al^3+^ exhibited little effects on the stability of AprBcp. According to the previous studies, metal ions act as activator or inhibitor mainly depend on the specific target alkaline proteases (Contesini et al., 2018; Barzkar, 2020). The result of this study and some previous research found that Cu^2+^ is an inhibitor. Otherwise, the alkaline proteases from *B. safensis* RH12 and *Penicillium chrysogenum* X5 are activated by Cu^2+^ and the residual activities are upregulated by 34.2 and 5.3%, respectively, when treated with 1 or 5 mM Cu^2+^ (Omrane Benmrad et al., 2018; Rekik et al., 2019).

**Table 3:** Effects of different metal cations on the stability of AprBcp.

Metal ions	Residual activity (%)	
	1 mM	5 mM
Na^+^	92.6	95.3
K^+^	97.2	93.6
Cu^2+^	25.9	40.7
Mn^2+^	104.6	112.5
Mg^2+^	103.2	108.6
Co^2+^	95.2	90.1
Al^3+^	97.9	90.5
Zn^2+^	94.1	95.2
Ca^2+^	107.2	115.3

### Substrate specificity profile of AprBcp

The substrate preferences of AprBcp were characterized with various natural proteinaceous substrates (Table 4). AprBcp exhibited the highest activity toward casein, followed by soybean protein isolate (61.2%) and bovine serum albumin (46.2%). Furthermore, AprBcp also displayed little activity against keratin and chicken egg albumin.

**Table 4:** The substrate specificity of AprBcp.

Substrates	Residual activity (%)
Casein	100
Soybean protein isolate	61.2
Bovine serum albumin	46.2
Keratin	8.6
Chicken egg albumin	11.3

### Effect of the enzymatic hydrolysis of soybean dregs on the protein recovery

**Figure 5:**
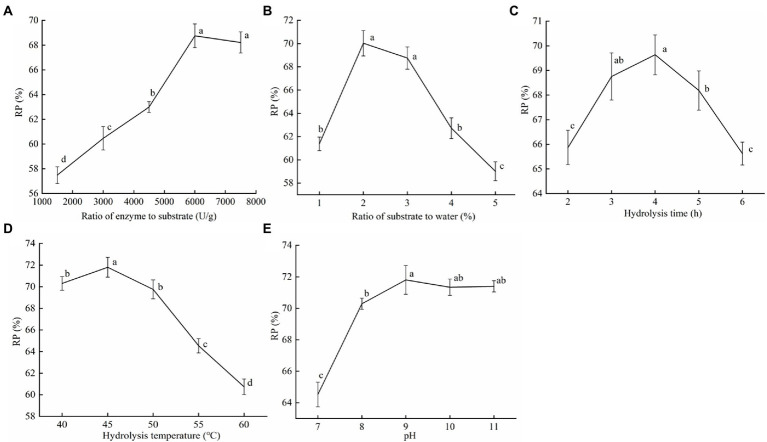
The effect of the proteinase AprBcp enzymatic hydrolysis of soybean dregs on the protein recovery. **(A)** Ratio of enzyme to substrate. **(B)** Ratio of substrate to water. **(C)** Hydrolysis time. **(D)** Hydrolysis temperature. **(E)** pH. Different lower case letters indicate significant differences (*p* < 0.05).

To search for the effect of the proteinase AprBcp enzymatic hydrolysis of soybean dregs on the protein recovery, single-factor experiments were carried out (Figure 5). The protein recovery (RP) gradually increased with the increase in the ratio of enzyme to substrate and leveled off after 2,000 U/g (Figure 5A). Therefore, the selected ratio of enzyme to substrate was 2,000 U/g. In addition, as ratio of substrate to water increased, the RP first increased and then decreased, reaching the maximum value at 2%, however, with no significant difference between 2 and 3% (Figure 5B). Considering the improvement of industrial production efficiency, ratio of substrate to water = 3% was selected. As shown in Figure 5C, when the hydrolysis time reached 4 h, the RP was the highest (69.64%). But, in industrial production, shorter enzymatic digestion time can prevent the growth of stray bacteria and reduce reaction costs. Thus, 3 h was selected as the hydrolysis time. Temperature is an important index affecting protease activity. The optimal temperature for enzymolysis of soybean dregs by proteinase AprBcp was 45°C, which was close to our above study (Figure 5D). When pH was 9.0, proteinase AprBcp exhibited the best hydrolysis efficiency in soybean dregs with a protein recovery of 71.81% (Figure 5E), which was higher than the results reported by Figueiredo et al. (2018).

## Conclusion

In conclusion, an alkaline protease (AprBcp) from *B. circulans* R1 was overexpressed, purified, and characterized. AprBcp belonged to S8 family peptidase. The maximum activity of recombinant strain Bs27 reached 165,870 U/ml. The purified AprBcp was most active at pH 10.0 and 60°C. Additionally, Ca^2+^ could as an activator to improve the stability of AprBcp. Furthermore, the recombinant AprBcp exhibited great potential application on recovery protein from soybean dregs. The excellent properties and overexpression of AprBcp will provide a basis for its further application on recovery protein from by-products from food and agriculture industry.

## Data availability statement

The datasets presented in this study can be found in online repositories. The names of the repository/repositories and accession number(s) can be found in the article/Supplementary material. GenBank ON733249.

## Author contributions

HC contributed to gene clone, construct recombinant strain, and bioinformatics analysis of AprBcp and writing. JWu contributed to characterization, purification, and kinetic parameters of AprBcp. XH contributed to recovery of protein from soybean dregs by AprBcp. XF contributed to large-scale production of AprBcp. HJ contributed to project administration. LZ contributed to the funding acquisition. JWa contributed to promoter optimization, signal peptide optimization, and experiment planning. All authors contributed to the article and approved the submitted version.

## Funding

This work was supported by the Science and Technology Innovation Program of Hunan Province (2019TP1028, 2019SK2122, 2019NK4229, and 2022NK2039), Postgraduate Scientific Research Innovation Project of Hunan Province (CX20211281, CX20211282), the Natural Science Foundation of Hunan Province (2021JJ40514), Guangdong Provincial Key Laboratory of Aquatic Product Processing and Safety (GDPKLAPPS2203), the National Key R&D Program (2019YFD0902000), and the Guangdong Innovation Team of Seafood Green Processing Technology (2019KCXTD011).

## Conflict of interest

XF was employed by Shenzhen Shanggutang Food Development Co., Ltd. JWa was employed by Shenzhen Raink Ecology & Environment Co., Ltd.

The remaining authors declare that the research was conducted in the absence of any commercial or financial relationships that could be construed as a potential conflict of interest.

## Publisher’s note

All claims expressed in this article are solely those of the authors and do not necessarily represent those of their affiliated organizations, or those of the publisher, the editors and the reviewers. Any product that may be evaluated in this article, or claim that may be made by its manufacturer, is not guaranteed or endorsed by the publisher.

## Footnotes

^1^https://www.expasy.org/ (Accessed July 25, 2022).

^2^https://www.genome.jp/tools-bin/clustalw (Accessed July 25, 2022).

^3^https://espript.ibcp.fr/ESPript/cgi-bin/ESPript.cgi(Accessed July 25, 2022).
