# Supplementary material for: Overexpression of Bacillus circulans alkaline protease in Bacillus subtilis and its potential application for recovery of protein from soybean dregs
Source: Front Microbiol. 2022 Aug 26;13:968439. doi: 10.3389/fmicb.2022.968439 (PMC9459226; doi:10.3389/fmicb.2022.968439)
Supplement: Supplementary file 1 [file Data_Sheet_1.docx]

**Supplement Methods**

**1. Cultivation conditions**

1.1. 24-well plate cultivation

Different single colonies were picked into the wells of 24-well plate containing 1.6 mL fermentation medium 1 supplemented with 30 mg/L kanamycin, and then, incubated at 37 °C and 200 rpm for 24 h. Finally, the 24-well plates were subjected to centrifugation and supernatants were used in subsequent activity assays. The clones showing higher activities were checked by shaking flask fermentation.

1.2. Shake Flask Cultures

Different single colonies were initially inoculated into 10 mL LB medium supplemented with 30 mg/L kanamycin in 150 mL flasks and incubated at 37 °C until the OD_600_ reached 1.0. Then, 2 mL seed solutions were transferred into 100 mL fermentation medium 1 supplemented with 30 mg/L kanamycin in 500 mL flasks and incubated at 37 °C and 200 rpm for 48 h. Meanwhile, 1 mL culture was harvested every 24 h for alkaline protease assay.

1.3. Large scale cultivation on 7 and 50 bioreactor

The single recombinant strain with highest activity was initially inoculated into 50 mL fermentation medium 1 in 250 mL flasks and incubated at 37 °C and 200 rpm for 24 h. Then, 2 mL seed solutions were transferred into 100 mL fermentation medium 1 in 500 mL flasks and incubated at 37 °C and 200 rpm for 24 h. Finally, the seed solutions from 500 mL flasks were all transferred into 3 or 20 L fermentation medium 2 in 7 or 50 L bioreactor. The temperature was controlled at 37 °C and the pH was maintained at 6.9. For 7 L bioreactor, the agitation rate was set at 500 rpm and the aeration rate was 30 L/min. For 50 L bioreactor, the agitation rate was set at 500 rpm and the aeration rate was 40 L/min. As the fermentation time reached about 10 h, the fermentation medium 3 was added automatically according to the dissolved oxygen (DO) and content of residual sugar. The fermentation medium 3 was composed of 45% maltose, 15% corn steep liquor, 0.6% K_2_HPO_4_, 0.02% MgSO_4_ and 0.3% CaCl_2_. Generally, the level of dissolved oxygen and residual sugar were controlled at 15-30% and 0.6-1.1%, respectively. The content of residual sugar was determined by DNS methods. The enzyme activity of the supernatant was monitored throughout the cultivation.

**2. Chromosomal integration of bcp expression cassette**

The recombinant strain containing *bcp* expression cassette was picked into 10 mL of LBK medium and cultivated in 45 °C for 16 h for plasmid integration. Then, the culture solution was diluted for 10^4^ to 10^5^-fold, spread onto LB plates with kanamycin and incubated at 45 °C until the single colony was formed. Streak culture of single colony on LB plates with kanamycin and incubated at 45 °C for about 11 h and colony PCR was used to screen the single-crossover recombinants. The single-crossover recombinants were picked and cultivated in LB at 37 °C and 200 rpm for 6-generation to facilitate the second recombination and plasmid excision. The culture was then diluted for 10^5^ to 10^6^-fold, spread onto LB plates and incubated at 37 °C until the single colony was formed. The single colony was picked and dotted on LB plate with and without kanamycin simultaneously. The single colony formed only in LB plate was chosen for further colony PCR and confirmed by DNA sequencing.


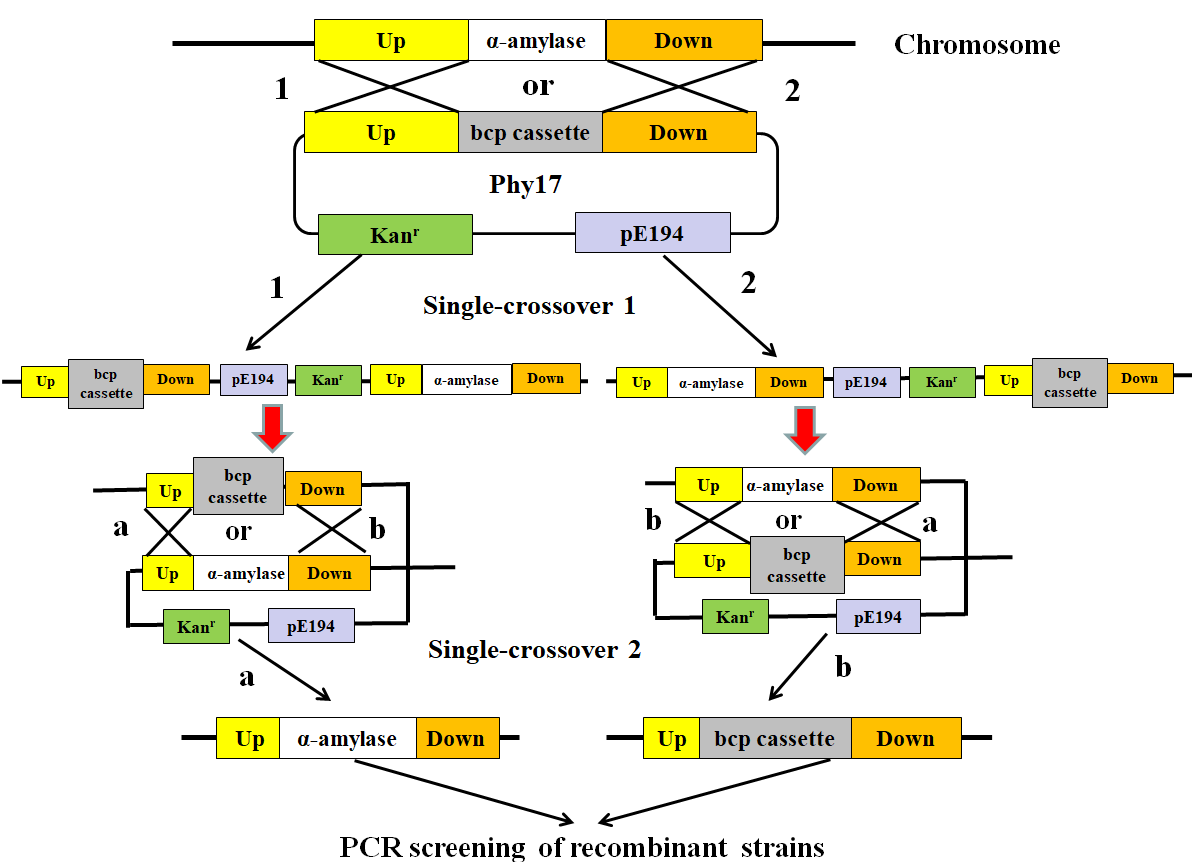


**Supplement Figure 1** Flowchart of chromosome integration of *bcp* expression cassette in *B. subtilis* 1285

Sequence of *bcp*

ATGAAGAAACCGTTGGGGAAAATTGTCGCAAGCGCCGCACTACTCCTTTCTGTTGCTTTTACTTCATCGATCGTATCGGCTGCTGAGGAAGCAAATGAAAAATATTTAATTGGCCTTAATGAGCAGGAAGCTGTCAGTGAGTTTGTAGAATCCATAGAGGCAAATGACGAGGTCGCCATTCTCTCTGAGGATGAGTCAGTCGAAATTGAATTGCTTCATGAGTTTGAAACGATTCCTGTTGTATCCGTTGAGTTAAGCCCAGAAGATGTGAAAGACCTTGAAAAAGATCCAGCGATTTCTTATACTGAAGAGGATATTGAAGTAACGATAATGGCGCAATCAGTGCCATGGGGAATTAGCCGTGTGCAAGCCCCAGCTGCCCATAACCGTGGATTGACAGGTTCTGGTGAAAAAGTTGCTGTCCTCGATACAGGTATTTCCACTCATCCAGACTTAAATATTCGTGGTGGCGCTAGCTTTGTACCAGGGGCACCATCCACTCAAGATGGGAATGGGCATGGCACGCATGTGGCCGGGACGATTGCTGCTTTAAACAATTCGATTGGCGTTCTTGGCGTAGCGCCGAGTGCGGAACTATACGCTGTTAATGTTTTAGGAGCCGACGGGAGAGGTGCAATCAGCTCGATTGCCCATGGGTTGGAATGGACAGGGAACAATAACATGCACGTTGCTAATTTAAGTTTAGGAAGCCCTTCGCCAAGTGCCACACTTGAGCAAGCTGTTAATAGCGCGACTTCTAGAGGCGTTCTTGTTGTAGCGGCATCTGGGAATTCAGGTGCAAGCTCAATCAGCTATCCGGCCCGTTATGCGAACGCAATGGCAGTCGGAGCTACTGACCAAAACAACAACCGCGCCAGCTTTTCACAGTATGGCGCAGGGCTTGACATTGTCGCACCAGGGGTAGGCGTGCAGAGCTCATACCCAGGTTCAACGTATGCCAGCTTAAACGGTACATCGATGGCTACTCCTCATGTTGCAGGTGTCGCAGCCCTTGTTAAACACAAGAACCCATCTTGGTCCAATACACAAATCCGCAACCATCTAGAGAATACGGCAACGAGCTTAGGAAGCACGAACTTGTATGTAAGCGGACTTGTCAAAGCAGAAGCGGCAACACGCTAA

**Supplement Table 1** Primers used in this study

| PCR products | Sequences of primer (5’-3’) |
| --- | --- |
| *bcp* | *bcp-*fw: ATGAAGAAACCGTTGGGGAAA |
|  | *bcp*-rev: TTAGCGTGTTGCCGCTTCTGC |
| *bcp* ligated to pHY | *bcp-*fw1: CGGTACCATTATACGGCCGATGAAGAAACCGTTGGGGAA |
|  | *bcp*-rev1: GTGATGATGGTGATGTCTAGATTAGCGTGTTGCCGCTTC |
| P_cry3A_ | P_cry3A_-fw: TAAAAACTGGTCTGAACGCGTTCGAAACGTAAGATGAAA |
|  | P_cry3A_-rev: CCAACGGTTTCTTCATCGGCCGCTTTTCATAATACATAATT |
| P_Blapr_ | P_Blapr_-fw: TAAAAACTGGTCTGAACGCGTACGCCTTTCACATGAGCT |
|  | P_Blapr_-rev: CCAACGGTTTCTTCATCGGCCGTACTCACTCTCCTCCGTT |
| P_Baamy_ | P_Baamy_-fw: TAAAAACTGGTCTGAACGCGTGCCCCGCACATACGAAAAG |
|  | P_Baamy_-rev: CCAACGGTTTCTTCATCGGCCGGTTTCCTCTCCCTCTCAT |
| P_Bsnpr_ | P_Bsnpr_-fw: TAAAAACTGGTCTGAACGCGTCAGCAAGGTCGAACGTATA |
|  | P_Bsnpr_-rev: CCAACGGTTTCTTCATCGGCCGAATAAATCCCCCTTTTTG |
| P_Bsamy_ | P_Bsamy_-fw: TAAAAACTGGTCTGAACGCGTCCGAGAATGGACACCAAA |
|  | P_Bsamy_-rev: CCAACGGTTTCTTCATCGGCCGTCTTGACACTCCTTATTT |
| P_HpaII_ | P_HpaII_-fw: TAAAAACTGGTCTGAACGCGTGGGCGCGATTGCTGAATAA |
|  | P_HpaII_-rev: CCAACGGTTTCTTCATCGGCCGATGTAAATCGCTCCTTTTT |
| P_gsiB_ | P_gsiB_ -fw: TAAAAACTGGTCTGAACGCGTGATCAAGACCGTACATAT |
|  | P_gsiB_ -rev: CCAACGGTTTCTTCATCGGCCGTTTGAATTCCTCCTTTAAT |
| P_Bsapr_ | P_Bsapr_-fw: TAAAAACTGGTCTGAACGCGTTATTTCTTCCTCCCTCTCA |
|  | P_Bsapr_-rev: CCAACGGTTTCTTCATCGGCCGTCTTTACCCTCTCCTTTTA |
| P_09_ | P_09_-fw: TAAAAACTGGTCTGAACGCGTGTCACAATGCGCCATCAA |
|  | P_09_-rev: CCAACGGTTTCTTCATCGGCCGTGATCCTTCCTCCTTTGT |
| The fragment for dual promoter | pHY-fw: TCGAAACGTAAGATGAAA |
|  | pHY-rev: ACGCGTTCAGACCAGTTTT |
| P_Bsamy-cry3A_ | P_Bsamy-cry3A_-fw: AAACTGGTCTGAACGCGTCCGAGAATGGACACCAAA |
|  | P_Bsamy-cry3A_-rev: TTTCATCTTACGTTTCGATCTTGACACTCCTTATTT |
| P_Baamy-cry3A_ | P_Baamy-cry3A_-fw: AAACTGGTCTGAACGCGTGCCCCGCACATACGAAAA |
|  | P_Baamy-cry3A_-rev: TTTCATCTTACGTTTCGAGTTTCCTCTCCCTCTCAT |
| P_Bsapr-cry3A_ | P_Bsapr-cry3A_-fw: AAACTGGTCTGAACGCGTTATTTCTTCCTCCCTCTC |
|  | P_Bsapr-cry3A_-rev: GTTTCCTCTCCCTCTCATTCTTTACCCTCTCCTTTT |
| P_HpaII-cry3A_ | P_HpaII-cry3A_-fw: AAACTGGTCTGAACGCGTGGGCGCGATTGCTGAATA |
|  | P_HpaII-cry3A_-rev: GTTTCCTCTCCCTCTCATATGTAAATCGCTCCTTTT |
| The fragment for signal peptide | pHY-S-fw: GCTGAGGAAGCAAAAGAAAAAT |
|  | pHY-S-rev: CGGCCGCTTTTCATAATACAT |
| SP_Bsamy_ | SP_Bsamy_-fw: ATTATGAAAAGCGGCCGATGTTTGCAAAACGATTCAAAAC CTCTTTACTGCCGTTATTCGCTGGATT |
|  | SP_Bsamy_-rev: TTCTTTTGCTTCCTCAGCAGCACTCGCAGCCGCCGGTCCTGC CAGAACCAAATGAAACAGCAATAAAAATCC |
| SP_Bsapr_ | SP_Bsapr_-fw: ATTATGAAAAGCGGCCGGTGAGAAGCAAAAAATTGTGGATC AGCTTGTTGTTTGCGTTAACGTTAA |
|  | SP_Bsapr_-rev: TTCTTTTGCTTCCTCAGCAGCCTGCGCAGACATGTTGCTGAAC GCCATCGTAAAGATTAACGTTAAC |
| SP_Bsnpr_ | SP_Bsnpr_-fw: ATTATGAAAAGCGGCCGGTGGGTTTAGGTAAGAAATTGTCT GTTGCTGTCGCTGCTTCGTTTAT |
|  | SP_Bsnpr_-rev: TTCTTTTGCTTCCTCAGCAGCCTGAACACCTGGCAGGCTGA TTGATAAACTCATAAACGAAGCAGCG |
| SP_Bschi_ | SP_Bschi_-fw: ATTATGAAAAGCGGCCGATGAAAAAAGTGTTTTCAAACAAA AAGTTTCTCGTTTTTTCTTTCATTTTTGCGA |
|  | SP_Bschi_-rev: TTCTTTTGCTTCCTCAGCGGCTTTTGCACTTTCCCCATTAAAA AAAGACAGACTTAAAATCATCGCAAAAATGA |
| SP_DacB_ | SP_DacB_-fw: ATTATGAAAAGCGGCCGATGCGCATTTTCAAAAAAGCAGTA TTCGTGATCATGATTTCTTTTC |
|  | SP_DacB_-rev: TTCTTTTGCTTCCTCAGC AGCATGTGCTGTATTCACATTTACGGTTGCAATAAGAAAAGAAAT |
| SP_Vpr_ | SP_Vpr_-fw: ATTATGAAAAGCGGCCGTTGAAAAAGGGGATCATTCGCTTTCT GCTTGTAAGTTTCGTCTTATTTTTT |
|  | SP_Vpr_-rev: TTCTTTTGCTTCCTCAGCAGCCGGAGCTGCCTGAACGCCCGTA ATGCCTGTGGATAACGCAAAAAATAAG |
| SP_YncM_ | SP_YncM_-fw: ATTATGAAAAGCGGCCGATGGCGAAACCACTATCAAAAGGG GGAATTTTGGTGAAAAAAGTATTGATTGCAGGTGCAGTAGGAACAGCAG |
|  | SP_YncM_-rev: TTCTTTTGCTTCCTCAGCAGCGTCTGCCGCGGGTAAACCTGG TATACCTGATGAAAGGGTTCCGAAAAGAACTGCTGTTCCTACTGCACCTGCAAT |
| SP_Bsap_ | SP_Bsap_-fw: ATTATGAAAAGCGGCCGATGAAAAAGCTTTTGACTGTCATGA CGATGGCTGTTTTAACTGCCGGCACA |
|  | SP_Bsap_-rev: ATTATGAAAAGCGGCCGAGCGTGCGCGGCAGGGGTGACACT CTGTGCCGGCAAGAGCAGTGTGCCGGCAGT |

**Supplement Table 2** Sequences of promoters used in this study

| Promoters | Sequences of primer (5’-3’) |
| --- | --- |
| P_43_ | TGTCGACGTGCATGCAGGCCGGGGCATATGGGAAACAGCGCGGACGGAGCGGAATTTCCAATTTCATGCCGCAGCCGCCTGCGCTGTTCTCATTTGCGGCTTCCTTGTAGAGCTCAGCATTATTGAGTGGATGATTATATTCCTTTTGATAGGTGGTATGTTTTCGCTTGAACTTTTAAATACAGCCATTGAACATACGGTTGATTTAATAACTGACAAACATCACCCTCTTGCTAAAGCGGCCAAGGACGCTGCCGCCGGGGCTGTTTGCGTTTTTACCGTGATTTCGTGTATCATTGGTTTACTTATTTTTTTGCCAAAGCTGTAATGGCTGAAAATTCTTACATTTATTTTACATTTTTAGAAATGGGCGTGAAAAAAAGCGCGCGATTATGTAAAATATAAAGTGATAGCGGTACCATTATA |
| P_cry3A_ | TCGAAACGTAAGATGAAACCTTAGATAAAAGTGCTTTTTTTGTTGCAATTGAAGAATTATTAATGTTAAGCTTAATTAAAGATAATATCTTTGAATTGTAACGCCCCTCAAAAGTAAGAACTACAAAAAAAGAATACGTTATATAGAAATATGTTTGAACCTTCTTCAGATTACAAATATATTCGGACGGACTCTACCTCAAATGCTTATCTAACTATAGAATGACATACAAGCACAACCTTGAAAATTTGAAAATATAACTACCAATGAACTTGTTCATGTGAATTATCGCTTTATTTAATTTTCTCAATTCAATATATAATATGCCAATACATTGTTACAAGTAGAAATTAAGACACCCTTGATAGCCTTACTATACCTAACATGATGTAGTATTAAATGAATATGTAAATATATTTATGATAAGAAGCGACTTATTTATAATCATTACATATTTTTCTATTGGAATGATTAAGATTCCAATAGAATAGTGTATAAATTATTTATCTTGAAAGGAGGGATGCCTAAAAACGAAGAACATTAAAAACATATATTTGCACCGTCTAATGGATTTATGAAAAATCATTTTATCAGTTTGAAAATTATGTATTATGAAAAG |
| P_Bsamy_ | CCGAGAATGGACACCAAAGAAGAACTGCAAAAACGGGTGAAGCAGCAGCGAATAGAATCAATTGCGGTCGCCTTTGCGGTAGTGGTGCTTACGATGTACGACAGGGGGATTCCCCATACATTCTTCGCTTGGCTGAAAATGATTCTTCTTTTTATCGTCTGCGGCGGCGTTCTGTTTCTGCTTCGGTATGTAATTGTGAAGCTGGCTTACAGAAGAGCGGTAAAAGAAGAAATAAAAAAGAAATCATCTTGAAAAATAGATGGTTTCTTTTTTTGTTTGGAAAGCGAGGGAAGCGTTCACAGTTTCGGGCAGCTTTTTTTATAGGAACATTGATTTGTATTCACTCTGCCAAGTTGTTTTGATAGAGTGATTGTGATAATTTAAAATGTAAGCGTTAACAAAATTCTCCAGTCTTCACATCAGTTTGAAAGGAGGAAGCGGAAGAATGAAGTAAGAGGGATTTTTGACTCCGAAGTAAGTCTTCAAAAAATCAAATAAGGAGTGTCAAGA |
| P_Baamy_ | GCCCCGCACATACGAAAAGACTGGCTGAAAACATTGAGCCTTTGATGACTGATGATTTGGCTGAAGAAGTGGATCGATTGTTTGAGAAAAGAAGAAGACCATAAAAATACCTTGTCTGTCATCAGACAGGGTATTTTTTATGCTGTCCAGACTGTCCGCTGTGTAAAAATAAGGAATAAAGGGGGGTTGTTATTATTTTACTGATATGTAAAATATAATTTGTATAAGAAAATGAGAGGGAGAGGAAAC |
| P_Bsnpr_ | CAGCAAGGTCGAACGTATAAAACTTACCCTTTCCGCCATGATCACGCGGCATCAGCATATAGTGAAAAGCCGTCAGCAGCACATATCCGTATAACAAAAAATGCAGCAGCGGCAGCAGTTCTTTTCCGTCCTCTCTTAAGTAAGCGCTGGTGAAGTTTGTTGATTGCACCTGGTGAATAAGTTCAACAGACACTCCCGCCAGCAGCACAATCCGCAATATAACACCCGCCAAGAACATTGTGCGCTGCCGGTTTATTTTGGGATGATGCACCAAAAGATATAAGCCCGCCAGAACAACAATTGACCATTGAATCAGCAGGGTGCTTTGTCTGCTTAATATAAAATAACGTTCGAAATGCAATACATAATGACTGAATAACTCCAACACGAACAACAATCCTTTACTTCTTATTAAGGCCTCATTCGGTTAGACAGCGGACTTTTCAAAAAGTTTCAAGATGAAACAAAAATATCTCATCTTCCCCTTGATATGTAAAAAACATAACTCTTGAATGAACCACCACATGACACTTGACTCATCTTGATATTATTCAACAAAAACAAACACAGGACAATACTATCAATTTTGTCTAGTTATGTTAGTTTTTGTTGAGTATTCCAGAATGCTAGTTTAATATAACAATATAAAGTTTTCAGTATTTTCAAAAAGGGGGATTTATT |
| P_HpaII_ | GGGCGCGATTGCTGAATAAAAGATACGAGAGACCTCTCTTGTATCTTTTTTATTTTGAGTGGTTTTGTCCGTTACACTAGAAAACCGAAAGACAATAAAAATTTTATTCTTGCTGAGTCTGGCTTTCGGTAAGCTAGACAAAACGGACAAAATAAAAATTGGCAAGGGTTTAAAGGTGGAGATTTTTTGAGTGATCTTCTCAAAAAATACTACCTGTCCCTTGCTGATTTTTAAACGAGCACGAGAGCAAAACCCCCCTTTGCTGAGGTGGCAGAGGGCAGGTTTTTTTGTTTCTTTTTTCTCGTAAAAAAAAGAAAGGTCTTAAAGGTTTTATGGTTTTGGTCGGCACTGCCGACAGCCTCGCAGAGCACACACTTTATGAATATAAAGTATAGTGTGTTATACTTTACTTGGAAGTGGTTGCCGGAAAGAGCGAAAATGCCTCACATTTGTGCCACCTAAAAAGGAGCGATTTACAT |
| P_gsiB_ | GATCAAGACCGTACATATAAGAATGTCGCTTCTCAAATCCAAGGCTGGCGAGAAGTCGTTTTGGGCTATCGAGACACGTTTGGCTGGAAAAAACTTTTCCAGATAGTGCCGGTTGCCGGAATGGTTTTTGGCGCCGCTGCCAATCGCTCAACATTAAACGACATTACCGAGACAGGCATGATGCTGTACAAAAAGAGGCGCATTCTTGAACGACTGAAAGAAACAGAACGAGAGATGGAATAGCAGAAAGCAGACGGACACCGCGATCCGCCTGCTTTTTTTAGTGGAAACATACCCAATGTGTTTTGTTTGTTTAAAAGAATTGTGAGCGGGAATACAACAACCAACACCAATTAAAGGAGGAATTCAAA |
| P_Bsapr_ | TATTTCTTCCTCCCTCTCAATAATTTTTTCATTCTATCCCTTTTCTGTAAAGTTTATTTTTCAGAATACTTTTATCATCATGCTTTGAAAAAATATCACGATAATATCCATTGTTCTCACGGAAGCACACGCAGGTCATTTGAACGAATTTTTTCGACAGGAATTTGCCGGGACTCAGGAGCATTTAACCTAAAAAAGCATGACATTTCAGCATAATGAACATTTACTCATGTCTATTTTCGTTCTTTTCTGTATGAAAATAGTTATTTCGAGTCTCTACGGAAATAGCGAGAGATGATATACCTAAATAGAGATAAAATCATCTCAAAAAAATGGGTCTACTAAAATATTATTCCATCTATTACAATAAATTCACAGAATAGTCTTTTAAGTAAGTCTACTCTGAATTTTTTTAAAAGGAGAGGGTAAAGA |
| P_Blapr_ | ACGCCTTTCACATGAGCTGATTTCATATCTTACACCCGTTTCTGTATGCGATATATTGCATATTTTAATAGATGATCGACTAGGCCGCAACCTCCTTCGGCAAAAAATGATCTCATAAAATAAATGAATAGTATTTTCATAAAATGAATCAGACGAAGCAATCTCCTGTCATTCACGGACCCCGGGACCTCTTTCCCTGCCAGGTTGAAGCGGTCTATTCATACTTTCGAACCGAATATTTTTCTAAAACAGTTATTAATAACCAATAAATTTAAATTGGCCGTTCAAAAAAATGGGTCTACCATATAATTCATTTTTTTTCTATAATAAATTAACAGAATAATTGGAATAGAGTATATTATTCTTCTATTTCAATTATTCTGAATAAAACGGAGGAGAGTGAGTA |
| P_09_ | GTCACAATGCGCCATCAAACCGTTGACAAGCGTCCCCGTCAGATGGCCGGGAGCCGGATGAACCACCATTCCGCGCGGCTTGTTGACGACAAGAACGTCCTGATCTTATTATAATATAAGCAAAAAACTCATAAAAAGGAAAAGCATTGACCTGAAAACTTATCGGTAAAGTATGATATAATACAAAAAGACCGATTAGAGGGGAGAGAGGAAACAAAGGAGGAAGGATCA |
| P_Bsapr-cry3A_ | TATTTCTTCCTCCCTCTCAATAATTTTTTCATTCTATCCCTTTTCTGTAAAGTTTATTTTTCAGAATACTTTTATCATCATGCTTTGAAAAAATATCACGATAATATCCATTGTTCTCACGGAAGCACACGCAGGTCATTTGAACGAATTTTTTCGACAGGAATTTGCCGGGACTCAGGAGCATTTAACCTAAAAAAGCATGACATTTCAGCATAATGAACATTTACTCATGTCTATTTTCGTTCTTTTCTGTATGAAAATAGTTATTTCGAGTCTCTACGGAAATAGCGAGAGATGATATACCTAAATAGAGATAAAATCATCTCAAAAAAATGGGTCTACTAAAATATTATTCCATCTATTACAATAAATTCACAGAATAGTCTTTTAAGTAAGTCTACTCTGAATTTTTTTAAAAGGAGAGGGTAAAGATCGAAACGTAAGATGAAACCTTAGATAAAAGTGCTTTTTTTGTTGCAATTGAAGAATTATTAATGTTAAGCTTAATTAAAGATAATATCTTTGAATTGTAACGCCCCTCAAAAGTAAGAACTACAAAAAAAGAATACGTTATATAGAAATATGTTTGAACCTTCTTCAGATTACAAATATATTCGGACGGACTCTACCTCAAATGCTTATCTAACTATAGAATGACATACAAGCACAACCTTGAAAATTTGAAAATATAACTACCAATGAACTTGTTCATGTGAATTATCGCTTTATTTAATTTTCTCAATTCAATATATAATATGCCAATACATTGTTACAAGTAGAAATTAAGACACCCTTGATAGCCTTACTATACCTAACATGATGTAGTATTAAATGAATATGTAAATATATTTATGATAAGAAGCGACTTATTTATAATCATTACATATTTTTCTATTGGAATGATTAAGATTCCAATAGAATAGTGTATAAATTATTTATCTTGAAAGGAGGGATGCCTAAAAACGAAGAACATTAAAAACATATATTTGCACCGTCTAATGGATTTATGAAAAATCATTTTATCAGTTTGAAAATTATGTATTATGAAAAG |
| P_Baamy-cry3A_ | GCCCCGCACATACGAAAAGACTGGCTGAAAACATTGAGCCTTTGATGACTGATGATTTGGCTGAAGAAGTGGATCGATTGTTTGAGAAAAGAAGAAGACCATAAAAATACCTTGTCTGTCATCAGACAGGGTATTTTTTATGCTGTCCAGACTGTCCGCTGTGTAAAAATAAGGAATAAAGGGGGGTTGTTATTATTTTACTGATATGTAAAATATAATTTGTATAAGAAAATGAGAGGGAGAGGAAACTCGAAACGTAAGATGAAACCTTAGATAAAAGTGCTTTTTTTGTTGCAATTGAAGAATTATTAATGTTAAGCTTAATTAAAGATAATATCTTTGAATTGTAACGCCCCTCAAAAGTAAGAACTACAAAAAAAGAATACGTTATATAGAAATATGTTTGAACCTTCTTCAGATTACAAATATATTCGGACGGACTCTACCTCAAATGCTTATCTAACTATAGAATGACATACAAGCACAACCTTGAAAATTTGAAAATATAACTACCAATGAACTTGTTCATGTGAATTATCGCTTTATTTAATTTTCTCAATTCAATATATAATATGCCAATACATTGTTACAAGTAGAAATTAAGACACCCTTGATAGCCTTACTATACCTAACATGATGTAGTATTAAATGAATATGTAAATATATTTATGATAAGAAGCGACTTATTTATAATCATTACATATTTTTCTATTGGAATGATTAAGATTCCAATAGAATAGTGTATAAATTATTTATCTTGAAAGGAGGGATGCCTAAAAACGAAGAACATTAAAAACATATATTTGCACCGTCTAATGGATTTATGAAAAATCATTTTATCAGTTTGAAAATTATGTATTATGAAAAG |
| P_Bsamy-cry3A_ | CCGAGAATGGACACCAAAGAAGAACTGCAAAAACGGGTGAAGCAGCAGCGAATAGAATCAATTGCGGTCGCCTTTGCGGTAGTGGTGCTTACGATGTACGACAGGGGGATTCCCCATACATTCTTCGCTTGGCTGAAAATGATTCTTCTTTTTATCGTCTGCGGCGGCGTTCTGTTTCTGCTTCGGTATGTAATTGTGAAGCTGGCTTACAGAAGAGCGGTAAAAGAAGAAATAAAAAAGAAATCATCTTGAAAAATAGATGGTTTCTTTTTTTGTTTGGAAAGCGAGGGAAGCGTTCACAGTTTCGGGCAGCTTTTTTTATAGGAACATTGATTTGTATTCACTCTGCCAAGTTGTTTTGATAGAGTGATTGTGATAATTTAAAATGTAAGCGTTAACAAAATTCTCCAGTCTTCACATCAGTTTGAAAGGAGGAAGCGGAAGAATGAAGTAAGAGGGATTTTTGACTCCGAAGTAAGTCTTCAAAAAATCAAATAAGGAGTGTCAAGATCGAAACGTAAGATGAAACCTTAGATAAAAGTGCTTTTTTTGTTGCAATTGAAGAATTATTAATGTTAAGCTTAATTAAAGATAATATCTTTGAATTGTAACGCCCCTCAAAAGTAAGAACTACAAAAAAAGAATACGTTATATAGAAATATGTTTGAACCTTCTTCAGATTACAAATATATTCGGACGGACTCTACCTCAAATGCTTATCTAACTATAGAATGACATACAAGCACAACCTTGAAAATTTGAAAATATAACTACCAATGAACTTGTTCATGTGAATTATCGCTTTATTTAATTTTCTCAATTCAATATATAATATGCCAATACATTGTTACAAGTAGAAATTAAGACACCCTTGATAGCCTTACTATACCTAACATGATGTAGTATTAAATGAATATGTAAATATATTTATGATAAGAAGCGACTTATTTATAATCATTACATATTTTTCTATTGGAATGATTAAGATTCCAATAGAATAGTGTATAAATTATTTATCTTGAAAGGAGGGATGCCTAAAAACGAAGAACATTAAAAACATATATTTGCACCGTCTAATGGATTTATGAAAAATCATTTTATCAGTTTGAAAATTATGTATTATGAAAAG |
| P_HpaII-cry3A_ | GGGCGCGATTGCTGAATAAAAGATACGAGAGACCTCTCTTGTATCTTTTTTATTTTGAGTGGTTTTGTCCGTTACACTAGAAAACCGAAAGACAATAAAAATTTTATTCTTGCTGAGTCTGGCTTTCGGTAAGCTAGACAAAACGGACAAAATAAAAATTGGCAAGGGTTTAAAGGTGGAGATTTTTTGAGTGATCTTCTCAAAAAATACTACCTGTCCCTTGCTGATTTTTAAACGAGCACGAGAGCAAAACCCCCCTTTGCTGAGGTGGCAGAGGGCAGGTTTTTTTGTTTCTTTTTTCTCGTAAAAAAAAGAAAGGTCTTAAAGGTTTTATGGTTTTGGTCGGCACTGCCGACAGCCTCGCAGAGCACACACTTTATGAATATAAAGTATAGTGTGTTATACTTTACTTGGAAGTGGTTGCCGGAAAGAGCGAAAATGCCTCACATTTGTGCCACCTAAAAAGGAGCGATTTACATTCGAAACGTAAGATGAAACCTTAGATAAAAGTGCTTTTTTTGTTGCAATTGAAGAATTATTAATGTTAAGCTTAATTAAAGATAATATCTTTGAATTGTAACGCCCCTCAAAAGTAAGAACTACAAAAAAAGAATACGTTATATAGAAATATGTTTGAACCTTCTTCAGATTACAAATATATTCGGACGGACTCTACCTCAAATGCTTATCTAACTATAGAATGACATACAAGCACAACCTTGAAAATTTGAAAATATAACTACCAATGAACTTGTTCATGTGAATTATCGCTTTATTTAATTTTCTCAATTCAATATATAATATGCCAATACATTGTTACAAGTAGAAATTAAGACACCCTTGATAGCCTTACTATACCTAACATGATGTAGTATTAAATGAATATGTAAATATATTTATGATAAGAAGCGACTTATTTATAATCATTACATATTTTTCTATTGGAATGATTAAGATTCCAATAGAATAGTGTATAAATTATTTATCTTGAAAGGAGGGATGCCTAAAAACGAAGAACATTAAAAACATATATTTGCACCGTCTAATGGATTTATGAAAAATCATTTTATCAGTTTGAAAATTATGTATTATGAAAAG |

**Supplement Table 3** Sequences of signal peptides used in this study

| signal peptides | Sequences of primer (5’-3’) |
| --- | --- |
| **SP_Bsamy_** | ATGTTTGCAAAACGATTCAAAACCTCTTTACTGCCGTTATTCGCTGGATTTTTATTGCTGTTTCATTTGGTTCTGGCAGGACCGGCGGCTGCGAGTGCT |
| **SP_Bsapr_** | GTGAGAAGCAAAAAATTGTGGATCAGCTTGTTGTTTGCGTTAACGTTAATCTTTACGATGGCGTTCAGCAACATGTCTGCGCAGGCT |
| **SP_Bsnpr_** | GTGGGTTTAGGTAAGAAATTGTCTGTTGCTGTCGCTGCTTCGTTTATGAGTTTATCAATCAGCCTGCCAGGTGTTCAGGCT |
| **SP_Bschi_** | ATGAAAAAAGTGTTTTCAAACAAAAAGTTTCTCGTTTTTTCTTTCATTTTTGCGATGATTTTAAGTCTGTCTTTTTTTAATGGGGAAAGTGCAAAAGCC |
| **SP_DacB_** | ATGCGCATTTTCAAAAAAGCAGTATTCGTGATCATGATTTCTTTTCTTATTGCAACCGTAAATGTGAATACAGCACATGCT |
| **SP_Vpr_** | TTGAAAAAGGGGATCATTCGCTTTCTGCTTGTAAGTTTCGTCTTATTTTTTGCGTTATCCACAGGCATTACGGGCGTTCAGGCAGCTCCGGCT |
| **SP_YncM_** | ATGGCGAAACCACTATCAAAAGGGGGAATTTTGGTGAAAAAAGTATTGATTGCAGGTGCAGTAGGAACAGCAGTTCTTTTCGGAACCCTTTCATCAGGTATACCAGGTTTACCCGCGGCAGACGCT |
| **SP_Bsap_** | ATGAAAAAGCTTTTGACTGTCATGACGATGGCTGTTTTAACTGCCGGCACACTGCTCTTGCCGGCACAGAGTGTCACCCCTGCCGCGCACGCT |

**Supplement Table** **4** Comparison of all screened signal peptides used in this study

| Name | Amino acid sequence | Hydrophobicity (%) | N-region Charge |
| --- | --- | --- | --- |
| SP_Bsamy_ | MFAKRFKTSLLPLFAGFLLLFHLVLAGPAAASA | 72.7 | 3 |
| SP_Bsapr_ | MRSKKLWISLLFALTLIFTMAFSNMSAQA | 58.6 | 3 |
| SP_Bschi_ | MKKVFSNKKFLVFSFIFAMILSLSFFNGESAKA | 54.5 | 4 |
| SP_Bsnpr_ | MGLGKKLSVAVAASFMSLSISLPGVQA | 59.2 | 2 |
| SP_DacB_ | MRIFKKAVFVIMISFLIATVNVNTAHA | 66.7 | 3 |
| SP_Vpr_ | MKKGIIRFLLVSFVLFFALSTGITGVQAAPA | 64.5 | 3 |
| SP_YncM_ | MAKPLSKGGILVKKVLIAGAVGTAVLFGTLSSGIPGLPAADA | 59.5 | 4 |
| SP_Bsap_ | MKKLLTVMTMAVLTAGTLLLPAQSVTPAAHA | 64.5 | 2 |
| SP_bcp_ | MKKPLGKIVASTALLISVAFSSSIVSA | 63.1 | 2 |

**Supplement Table 5** Purification of AprBcp from culture supernatant

| Purification steps | Specific activity (U/mg) | Purification fold |
| --- | --- | --- |
| Supernatant | 27952 | 1 |
| Ultrafiltration | 51246 | 1.83 |
| HiTrap SP Fast Flow | 58621 | 2.09 |


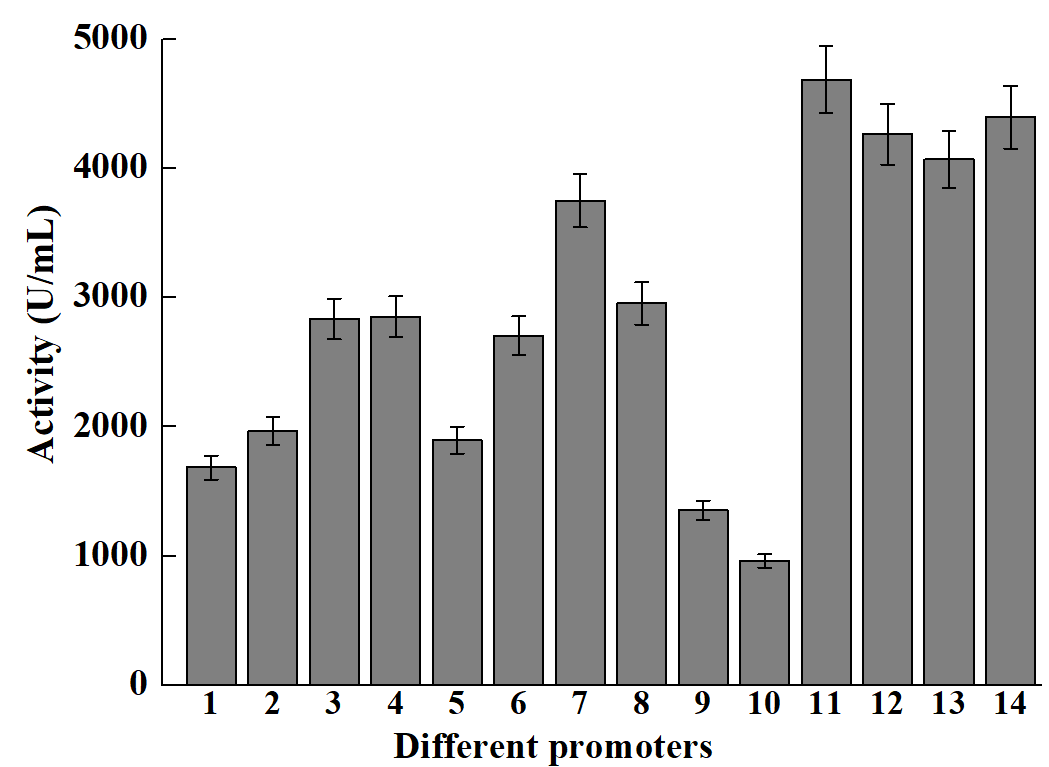


**Supplement Figure 2** The extracellular activities of strain (Bs 1-14) containing different promoters cultivated in 24-well plate. Number 1-14 represent promoter P_43_, P_gsiB_, P_Bsamy_, P_Baamy_, P_Bsnpr_, P_HpaII_, P_cry3A_, P_Bsapr_, P_Blapr_, P_09_, P_Bsapr-cry3A_, P_Baamy-cry3A_, P_Bsamy-cry3A_ and P_HpaII-cry3A_.


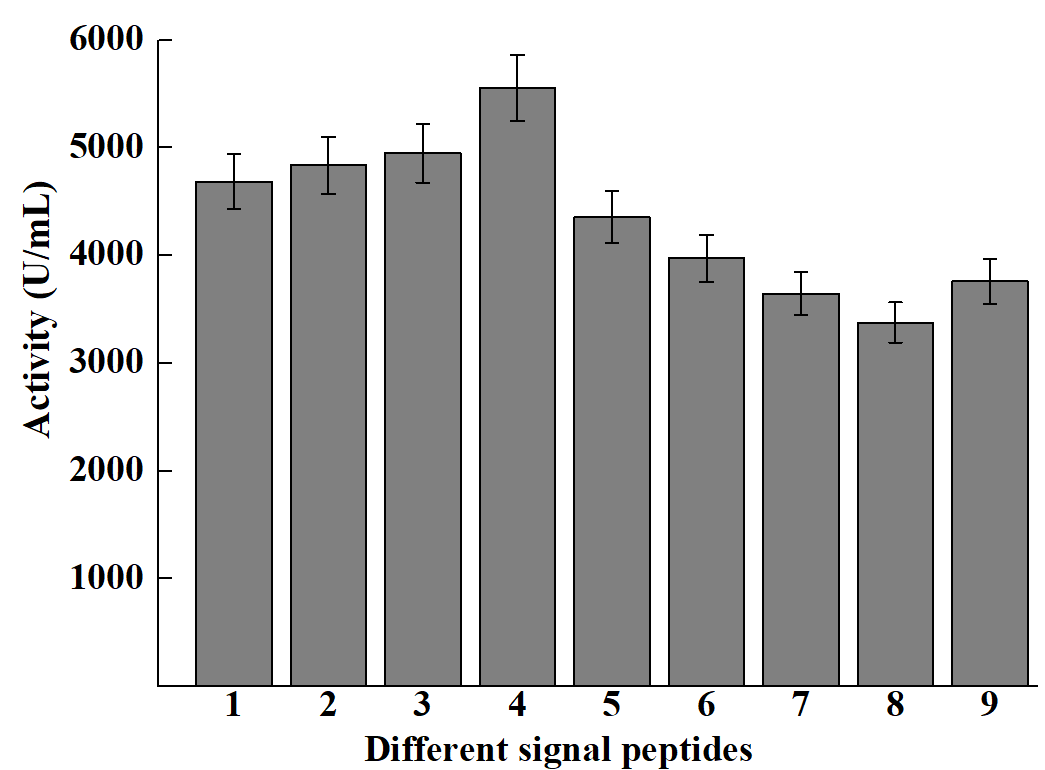


**Supplement Figure 3** The extracellular activities of strain containing different signal peptide cultivated in 24-well plate. Number 1-9 represent represent signal peptide SP_bcp_, SP_Bsamy_, SP_Bsapr_, SP_Bschi_, SP_Bsnpr_, SP_DacB_, SP_Vpr_, SP_YncM_ and SP_Bsap_.
